# ADHD and cardiometabolic risk profile in adults with type 2 diabetes: a longitudinal register-based study

**DOI:** 10.1136/bmjopen-2025-113372

**Published:** 2026-07-03

**Authors:** Zihan Dong, Shengxin Liu, Cecilia Lundholm, Isabell Brikell, Ralf Kuja-Halkola, Brian M D’Onofrio, Paul Lichtenstein, Agnieszka Butwicka, Soffia Gudbjörnsdottir, Henrik Larsson, Zheng Chang, Ebba Du Rietz

**Affiliations:** 1 Department of Medical Epidemiology and Biostatistics, Karolinska Institutet, Stockholm, Sweden; 2 Department of Global Public Health and Primary Care, University of Bergen, Bergen, Norway; 3 Department of Biomedicine, Aarhus Universitet, Aarhus, Denmark; 4 Department of Psychological and Brain Sciences, Indiana University, Bloomington, Indiana, USA; 5 Division of Mental Health Services, Akershus University Hospital, Lørenskog, Norway; 6 Institute of Clinical Medicine, University of Oslo, Oslo, Norway; 7 Department of Biostatistics and Translational Medicine, Medical University of Lodz, Łódź, Poland; 8 Department of Medical Sciences, Child and Adolescent Psychiatry, Uppsala University, Uppsala, Sweden; 9 Department of Molecular and Clinical Medicine, Sahlgrenska Academy, University of Gothenburg, Gothenburg, Sweden; 10 Swedish National Diabetes Register, Centre of Registers Vastra Gotaland, Gothenburg, Sweden; 11 School of Medical Sciences, Örebro universitet, Örebro, Sweden

**Keywords:** Attention Deficit Disorder with Hyperactivity, Diabetes Mellitus, Type 2, Body Mass Index, Lipid disorders, Smoking Reduction

## Abstract

**Abstract:**

**Objective:**

To investigate the association between attention-deficit/hyperactivity disorder (ADHD) and cardiometabolic risk profile at the time of type 2 diabetes (T2D) diagnosis and examine longitudinal changes in cardiometabolic measures following T2D diagnosis.

**Design and setting:**

A nationwide cohort study using linked Swedish health registers.

**Participants:**

Adults aged 18–65 years with a first recorded diagnosis of T2D between 1996 and 2020. ADHD, treated as a lifetime condition, was identified through diagnostic and prescription records.

**Outcome measures:**

The cardiometabolic risk profile, comprising nine clinical parameters and two behavioural factors, was assessed at T2D diagnosis and was tracked for up to 5 years post-diagnosis. Linear regression (β_ADHD_) and Poisson regression models with robust variance (risk ratios (RRs)) compared cardiometabolic risk factors at diagnosis between individuals with and without ADHD. Generalised estimating equations (β_ADHD*t_) assessed longitudinal changes in clinical parameters following the diagnosis.

**Results:**

Among 80 607 individuals with T2D, 1204 (1.5%) had an ADHD diagnosis. At T2D diagnosis, individuals with ADHD were younger and had statistically significantly higher body mass index (BMI) (β_ADHD_: 1.93 (95% CI 1.54 to 2.32)), triglycerides (β_ADHD_: 0.31 (95% CI 0.17 to 0.45)) and smoking prevalence (RR: 1.58 (95% CI 1.44 to 1.74)) compared with those without ADHD. Other cardiometabolic risk factors did not differ significantly. Over the subsequent 5 years, trajectories in cardiometabolic risk factors were broadly similar, except for a greater reduction in BMI in individuals with ADHD (β_ADHD*t_: −0.26 (95% CI −0.34 to −0.17)), independent of baseline BMI and glucose-lowering medications. After inverse probability of treatment weighting, the BMI reduction was substantially reduced and not significant (β_ADHD*t_: −0.05 (95% CI −0.17 to 0.07)).

**Conclusions:**

Among adults with newly diagnosed T2D, those with co-occurring ADHD had broadly similar cardiometabolic profiles to those without ADHD but presented at a younger age with a modestly higher BMI, triglycerides and smoking prevalence. Individuals with ADHD also showed a slightly greater reduction in BMI over time following T2D diagnosis. Despite modest overall cardiometabolic differences, incorporating ADHD status into preventive diabetes care may help identify a younger, more vulnerable subgroup who could benefit from targeted risk factor management.

**STRENGTHS AND LIMITATIONS OF THIS STUDY**

- The study used data from the Swedish National Diabetes Register, which prospectively collects detailed data on clinical parameters and behavioural factors covering the majority of individuals with type 2 diabetes in Sweden (87% coverage in 2019).
- The data were collected prospectively, minimising any recall bias.
- Psychiatric and somatic disorders were identified using International Classification of Diseases codes from the Swedish National Patient Register with high national coverage, reducing potential misclassification biases.
- The Swedish register data only includes individuals who visit healthcare facilities, potentially excluding those with less severe health conditions.
- Improved register coverage, diagnostic practices and attention-deficit/hyperactivity disorder (ADHD) awareness over time may lead to underreported ADHD in the older cohort.

## Introduction

 Attention-deficit/hyperactivity disorder (ADHD) is a neurodevelopmental condition characterised by persistent inattention, hyperactivity and impulsivity that interferes with functioning. ADHD is diagnosed in around 5% of school-aged children, and at least two-thirds of children with ADHD continue to have impairing symptoms in adulthood.^1^ Large, population-based studies show that adults with ADHD have increased risks of several adverse health outcomes, including metabolic and cardiovascular diseases (CVDs)^2 3^ and premature mortality.^4^ Emerging evidence also indicates a roughly twofold higher risk of type 2 diabetes (T2D), a common metabolic disorder characterised by chronic hyperglycaemia among individuals with ADHD.^2 5 6^

Despite the elevated risk of T2D among individuals with ADHD, it remains unclear how ADHD relates to the cardiometabolic risk profile in individuals with T2D. In the general population, ADHD has been linked to several cardiometabolic risk factors, such as higher body mass index (BMI)^7^,^9^ and blood pressure, increased risk of hypertension^59^,^11^ and unhealthy lifestyle behaviours, such as smoking^89 12^,^14^ and physical inactivity.^15 16^ However, research relating to other cardiometabolic risk factors, such as lipid profiles and glycaemic indices, remains limited and inconsistent.^10^,^1217^

Managing T2D, which typically involves controlling blood pressure, lipids, BMI and blood glucose,^22^ can be challenging for individuals with ADHD due to difficulties with organisation and planning, as effective neurocognitive skills are required to execute and comply with diabetes management (through medication, healthcare visits and maintaining a healthy lifestyle).^23^ Poorer glycaemic control and increased risk of diabetic complications have been observed in individuals with co-occurring type 1 diabetes (T1D) and ADHD.^24 25^ However, research on these parameters in individuals with co-occurring T2D and ADHD is limited. A small cross-sectional study (n=315) showed no association between ADHD-like symptoms and haemoglobin A1c (HbA1c), lipid profile and estimated glomerular filtration rate (eGFR) in individuals with T2D.^26^

Diabetes care guidelines emphasise the importance of considering co-occurring psychiatric conditions, such as schizophrenia and major depressive disorder.^27 28^ However, ADHD, an increasingly recognised diagnosis in adults, is not addressed in these guidelines. Emerging evidence indicated that individuals with co-occurring T2D and ADHD often constitute a younger subgroup of people with T2D.^26^ This group may warrant particular attention, as early-onset T2D is linked with higher risks of cardiovascular complications and mortality.^29^

Given the scarcity of research and clinical guidance on how ADHD influences the cardiometabolic risk profile in T2D, we conducted a nationwide Swedish register study to compare cardiometabolic risk profiles between adults with newly diagnosed T2D with and without ADHD. We assessed clinical parameters and behavioural factors at the time of T2D diagnosis and examined longitudinal changes thereafter, up to 5 years for clinical parameters and up to 2 years for behavioural factors.

## Methods

### Data sources and linkage

We linked nationwide Swedish registers using unique personal identification numbers^30^: the Total Population Register,^31^ the National Patient Register,^32^ the Prescribed Drug Register^33^ and the Swedish National Diabetes Register (details are in the online supplemental materials).^34^

### Study cohort

This cohort study included all individuals in Sweden aged 18–65 years with a first recorded diagnosis of T2D between 1996 and 2020. The T2D diagnosis was identified from the National Patient Register and National Diabetes Register (International Classification of Diseases, Tenth Revision (ICD-10) code E11). We excluded individuals who had no records of cardiometabolic measures of interest in the National Diabetes Register, had their first measurement after 1 month since T2D diagnosis (ie, baseline), had been prescribed glucose-lowering medication (Anatomical Therapeutic Chemical (ATC) code A10B) before or at the first measurement, had emigrated, had death records (data error) or had T1D diagnosis (ICD-8/9 code: 250 with age<18 years, or ICD-10: E10) before or at the first recorded T2D diagnosis (online supplemental figures S1 and S2).

### Exposure

The exposure was ADHD, which was regarded as a lifetime condition, meaning that the ADHD diagnosis could be recorded before or after the T2D diagnosis. Individuals were defined as having ADHD based on at least one ADHD diagnosis (ICD-9 or ICD-10: 314 /F90) record from the National Patient Register or ADHD medication dispensation (ATC codes N06BA04/N06BA01/N06BA02/N06BA12/N06BA09/C02AC02) record from the Prescribed Drug Register. Prior studies have demonstrated that this register-based ADHD definition in Sweden exhibits high specificity^35^ and that aetiological patterns remain consistent regardless of whether ADHD identification is based on clinical diagnoses or prescription records.^36^ Furthermore, in Sweden, only senior physicians specialised in psychiatry or neurology are permitted to prescribe medication for ADHD.^37^

### Outcomes and follow-up

The outcomes were the cardiometabolic risk profile (including clinical parameters and behavioural factors) measured during healthcare visits, recorded in the National Diabetes Register (online supplemental table S1). Clinical parameters included nine continuous variables: HbA1c (indicator for glycaemic control, measured in mmol/mol and converted to percentage (%) for dual reporting according to the National Glycohemoglobin Standard Programme (NGSP) standard)^38^; BMI (kg/m^2^); systolic and diastolic blood pressure (mm Hg); lipid profile (low-density lipoprotein cholesterol (LDLc, mmol/L), high-density lipoprotein cholesterol (HDLc, mmol/L), total cholesterol (mmol/L) and triglycerides (mmol/L)) and eGFR (mL/min/1.73 m^2^). Behavioural factors included two binary variables: smoking (yes/no) and physical activity level (low/high).

In baseline analyses, for each clinical parameter and behavioural factor, we included people who had a first measurement in the National Diabetes Register within the first month after T2D diagnosis. In longitudinal analyses, we included individuals with at least one follow-up measurement of clinical parameters recorded in the National Diabetes Register, and the clinical parameters were repeatedly examined over 5 years following T2D diagnosis (ie, the measurement window is from T2D diagnosis to end of study, 30 January 2023 or 5 years following T2D diagnosis, whichever came first (online supplemental figure S2)). To examine engagement in non-pharmacological intervention (smoking cessation, changing from low to high levels of physical activity), that is, sustained changes in unhealthy lifestyle behaviours, we studied the behavioural factors 2 years after T2D diagnosis in people who were recorded as smokers or having low levels of physical activity at T2D diagnosis (online supplemental figure S1).

### Covariates

Covariates were obtained from the National Patient Register and examined before or at T2D diagnosis. They included sex; calendar year and age at T2D diagnosis; psychiatric disorder history (anxiety disorders, autism spectrum disorder, bipolar disorder, conduct disorder, major depressive disorder, eating disorders, intellectual disability, personality disorders, schizophrenia and psychotic disorders and substance use disorders) and somatic disorder history (CVDs, obesity (clinically diagnosed obesity requiring specialised care),^39^ hyperlipidaemia and sleep disorders) (online supplemental table S2). Sex and each psychiatric and somatic disorder were included as binary variables, while year and age were treated as continuous variables.

### Statistical analyses

Covariate balance between the ADHD and non-ADHD groups was assessed using standardised mean differences (SMDs), with p values additionally reported to describe statistical differences between groups using the Wilcoxon rank-sum test and χ^2^ tests.

In baseline analyses, we applied linear regression for continuous variables and Poisson regression with robust variance for binary variables^40^ to estimate the association of ADHD and cardiometabolic risk profile at T2D diagnosis. For longitudinal analyses, we used generalised estimating equation (GEE) models to assess associations between ADHD and changes in clinical parameters over the 5 years following T2D diagnosis. An interaction term between ADHD and time (years) since T2D diagnosis (β_ADHD*t_) was included to estimate differences in annual rates of change over follow-up between groups. Time was modelled as a continuous variable to accommodate irregular measurement intervals. An exchangeable correlation structure accounted for within-individual correlations across repeated measurements, varying follow-up intervals and numbers of observations per individual, to estimate population-averaged longitudinal changes. Non-linear time effects were not modelled, as the irregular timing and frequency of routine clinical measurements supported a parsimonious linear specification. We used Poisson regression with robust variance to study the association between ADHD and improvements in behavioural factors 2 years after T2D diagnosis. Regression coefficients (β_ADHD_ in linear regression, β_ADHD*t_ in GEE) and risk ratios (RRs) with 95% CIs were reported. Associations were first adjusted for sex, age and calendar year at T2D diagnosis (Model 1) and then additionally adjusted for psychiatric (Model 2) and somatic (Model 3) disorder history. A false discovery rate-adjusted p-value threshold of 0.0001, generated using the Benjamini-Hochberg procedure,^41^ was used to infer statistical significance to account for multiple testing across the 72 models in the main analyses and reduce the likelihood of false-positive findings.

In stratified analyses, associations were examined by sex and birth cohort (individuals born 1930–1959 or 1960–2002) to explore differences in the links between ADHD and cardiometabolic risk profile. In sensitivity analyses, we first re-ran the main analyses solely using the ADHD diagnosis to define ADHD, minimising potential misclassification due to off-label use of ADHD medication. Second, we additionally adjusted for BMI in the main analyses to see if BMI could explain the associations between ADHD and other clinical parameters. Third, to examine whether ADHD medications influenced the change of clinical parameters following T2D diagnosis, we measured whether the individuals had taken any ADHD medication after T2D diagnosis and before the first follow-up measurement, defining ADHD status as categorical (no ADHD, ADHD without ADHD medication or ADHD with medication). To further account for the potential impact of severe psychiatric comorbidities with ADHD, we conducted sensitivity analyses by excluding individuals with a history of schizophrenia, bipolar disorder or substance use disorder. Additionally, to assess the influence of psychotropic medications on weight, we performed sensitivity analyses by adjusting for the use of antipsychotics, antidepressants and antiepileptics within 3 months prior to the T2D diagnosis. In another model, we specifically included second-generation antipsychotics as a separate variable, given their relatively stronger association with weight gain.^42^ Moreover, we conducted an additional sensitivity analysis by defining ADHD status within 5 years prior to the T2D diagnosis to capture more recent ADHD cases. To further address potential issues of imbalanced covariates between the ADHD and non-ADHD groups, we conducted a sensitivity analysis using inverse probability of treatment weighting (IPTW) based on propensity scores. The propensity scores were estimated using logistic regression, including demographic characteristics and relevant psychiatric and somatic comorbidities used in the fully adjusted model, and stabilised weights were used in the analyses. Stratified and sensitivity analyses were applied to the fully adjusted Model 3. Data management and analyses were performed using SAS (V.9.4, SAS Institute Inc.). We adhered to the Strengthening the Reporting of Observational Studies in Epidemiology (STROBE) guidelines for cohort studies.^43^

### Patient and public involvement

Patients and members of the public did not participate in the design, implementation, reporting or dissemination of this study.

## Results

### Cohort characteristics

We identified 261 214 adults aged 18–65 years with a first recorded T2D diagnosis between 1996 and 2020, of whom 80 607 met the inclusion criteria (online supplemental figure S1). A substantial imbalance in baseline covariates was observed between the ADHD and non-ADHD groups (table 1), with the exception of sex and CVDs; most differences were statistically significant. Individuals with ADHD were younger at T2D diagnosis (median 45.6 years (IQR: 36.1–53.0) vs 56.9 years (IQR: 49.9–61.8)) and more often diagnosed with T2D in recent years. The majority of individuals were female (ADHD: 60.0% vs non-ADHD: 58.6%). Individuals with ADHD had higher proportions of psychiatric and somatic comorbidities compared with those without ADHD (table 1).

**Table 1:** Demographic and clinical characteristics of study population at baseline, by ADHD status

Variables	ADHD group	Non-ADHD group	SMDs	P value
	(n=1 204)	(n=79 403)		
Age at first recorded T2D (years), median (IQR)	45.6 (36.1, 53.0)	56.9 (49.9, 61.8)	1.02	<0.001†
Year at first recorded T2D (years), median (IQR)	2015 (2012, 2018)	2012 (2009, 2016)	0.47	<0.001†
Female, n (%)	723 (60.0)	46 515 (58.6)	0.03*	0.32
Somatic comorbidities, n (%)				
Obesity‡	208 (17.3)	5 085 (6.4)	0.34	<0.001†
Cardiovascular diseases	307 (25.5)	22 769 (28.7)	0.07^*^	0.02†
Hyperlipidaemia	228 (18.9)	22 609 (28.5)	0.23	<0.001†
Sleep disorder	205 (17.0)	5 639 (7.1)	0.31	<0.001†
Psychiatric comorbidities, n (%)				
Anxiety disorder	239 (19.9)	1 950 (2.5)	0.57	<0.001†
Autism spectrum disorder	180 (15.0)	208 (0.3)	0.58	<0.001†
Bipolar disorder	163 (13.5)	926 (1.2)	0.49	<0.001†
Conduct disorder	17 (1.4)	31 (0.04)	0.16	<0.001†
Major depressive disorder	538 (44.7)	5 567 (7.0)	0.95	<0.001†
Eating disorders	19 (1.6)	95 (0.1)	0.16	<0.001†
Intellectual disability	73 (6.1)	497 (0.6)	0.31	<0.001†
Personality disorders	211 (17.5)	1 383 (1.7)	0.56	<0.001†
Schizophrenia and psychotic disorders	96 (8.0)	1 911 (2.4)	0.25	<0.001†
Substance use disorders	369 (30.6)	4 028 (5.1)	0.71	<0.001†

* An SMD before IPTW. SMD<0.1 was indicative of adequate balance between ADHD and non-ADHD groups.

† p<0.05 was indicative of statistically significant differences regarding the characteristics between ADHD and non-ADHD groups.

‡ Clinically diagnosed obesity requiring specialised care.

ADHD, attention-deficit/hyperactivity disorder; IQR, interquartile range; SMDs, standardized mean differences; T2D, type 2 diabetes.

During follow-up, the frequency and timing of clinical parameter measurements were comparable between groups (online supplemental table S3 and figure S3). Most individuals received glucose-lowering medication during follow-up (ADHD: 77.5% vs non-ADHD: 72.2%), and the median follow-up window was similar (5.0 years in both groups).

### ADHD and cardiometabolic profile at T2D diagnosis

Descriptive statistics indicated that at the first recorded T2D diagnosis, the ADHD group exhibited a slightly more detrimental cardiometabolic profile, including higher levels of HbA1c, diastolic blood pressure, BMI, total cholesterol, triglycerides and LDLc and lower levels of HDLc compared with the non-ADHD group. Compared with the non-ADHD group, a higher proportion of individuals with ADHD were smokers (36% vs 21%) and reported low physical activity levels (55% vs 48%) at T2D diagnosis (table 2). Conversely, the ADHD group exhibited lower mean systolic blood pressure and higher eGFR levels, which are indicators of more favourable cardiometabolic profiles (table 2).

**Table 2:** Association between ADHD and cardiometabolic risk profile at type 2 diabetes diagnosis

Clinical parameters	N		Mean (SD)		Beta coefficients (95% CI)		
	ADHD	non-ADHD	ADHD	non-ADHD	Model 1	Model 2	Model 3
HbA1c (%)	1 083	71 665	7.23 (1.77)	6.98 (1.58)	−0.01 (−0.11 to 0.08)	0.002 (−0.10 to 0.10)	0.01 (−0.09 to 0.11)
HbA1c (mmol/mol)			55.51 (19.32)	52.78 (17.30)	−0.14 (−1.17 to 0.90)	0.02 (−1.07 to 1.12)	0.13 (−0.96 to 1.22)
Systolic blood pressure (mm Hg)	974	67 117	132.20 (15.35)	136.12 (16.69)	0.09 (−0.95 to 1.13)	1.08 (−0.02 to 2.18)	1.07 (−0.03 to 2.17)
Diastolic blood pressure (mm Hg)	976	67 027	83.19 (10.51)	82.35 (10.09)	0.24 (−0.40 to 0.88)	0.39 (−0.28 to 1.07)	0.39 (−0.29 to 1.06)
BMI (kg/m^2^)	869	58 884	35.43 (7.24)	31.88 (5.95)	1.93 (1.54 to 2.32)*	1.32 (0.91 to 1.74)*	0.92 (0.53 to 1.31)*
Total cholesterol (mmol/L)	783	55 886	5.42 (1.35)	5.33 (1.19)	0.11 (0.02 to 0.19)	0.05 (−0.04 to 0.14)	0.07 (−0.01 to 0.16)
LDLc (mmol/L)	698	51 061	3.36 (1.09)	3.31 (1.02)	0.02 (−0.06 to 0.09)	0.01 (−0.07 to 0.09)	0.03 (−0.05 to 0.11)
HDLc (mmol/L)	708	51 035	1.08 (0.33)	1.19 (0.36)	−0.02 (−0.05 to 0.00)	−0.01 (−0.04 to 0.01)	−0.01 (−0.04 to 0.02)
Triglycerides (mmol/L)	668	49 359	2.72 (2.36)	2.15 (1.79)	0.31 (0.17 to 0.45)*	0.14 (0.001 to 0.29)	0.14 (−0.01 to 0.28)
eGFR (mL/min/1.73 m^2^)	938	62 383	100.16 (25.49)	91.44 (22.41)	−1.26 (−2.62 to 0.10)	−0.79 (−2.22 to 0.64)	−0.65 (−2.08 to 0.77)

Behavioural factors	n (%)		Risk ratio (95% CI)		
	ADHD	Non-ADHD	Model 1	Model 2	Model 3
Smoking	n=850	n=60 420			
	307 (36)	12 941 (21)	1.58 (1.44 to 1.74)*	1.13 (1.03 to 1.25)	1.14 (1.04 to 1.26)
Low physically active	n=640	n=44 604			
	351 (55)	21 264 (48)	1.08 (1.00 to 1.16)	1.00 (0.93 to 1.08)	0.98 (0.91 to 1.06)

Target management levels for clinical parameters in individuals with type 2 diabetes: HbA1c: <7% (53 mmol/mol), diastolic blood pressure: <80 mm Hg, systolic blood pressure: <130 mm Hg, BMI: <25 kg/m², total cholesterol: <4 mmol/L, LDLc: <2.6 mmol/L, HDLc: >1.0 mmol/L, triglycerides: <1.7 mmol/L, eGFR: ≥90 mL/min/1.73 m².

n: For clinical parameters, refers to the number of individuals with corresponding measurements; for behavioural factors, refers to the number and proportion of individuals who are smokers or have low physical activity levels among those whose smoking status or physical activity levels were measured for the ADHD group and non-ADHD group at type 2 diabetes diagnosis. Model 1: adjusted for calendar year, age and sex at type 2 diabetes diagnosis; Model 2: additionally adjusted for other psychiatric disorders’ history; Model 3: additionally adjusted for somatic disorders’ history.

* Statistically significant at a false discovery rate-corrected p-value<0.0001.

ADHD, attention-deficit/hyperactivity disorder; BMI, body mass index; eGFR, estimated glomerular filtration rate; HbA1c, haemoglobin A1C; HDLc, high-density lipoprotein cholesterol; LDLc, low-density lipoprotein cholesterol.

Statistical analyses, after adjusting for sex, age and calendar year at T2D, showed that individuals with T2D and ADHD had statistically significantly higher BMI (β_ADHD_: 1.93 (95% CI 1.54 to 2.32); table 2) and triglycerides (β_ADHD_: 0.31 (95% CI 0.17 to 0.45); table 2) than those without ADHD. The association for BMI was attenuated but remained significant after additionally adjusting for psychiatric and somatic disorder history (β_ADHD_: 0.92 (95% CI 0.53 to 1.31); table 2). Individuals with ADHD were also statistically significantly more likely to be smokers (RR: 1.58 (95% CI 1.44 to 1.74); table 2)). The remaining nine cardiometabolic risk factors did not significantly differ between individuals with and without ADHD.

### ADHD and change of cardiometabolic profile following T2D diagnosis

**Figure 1:**
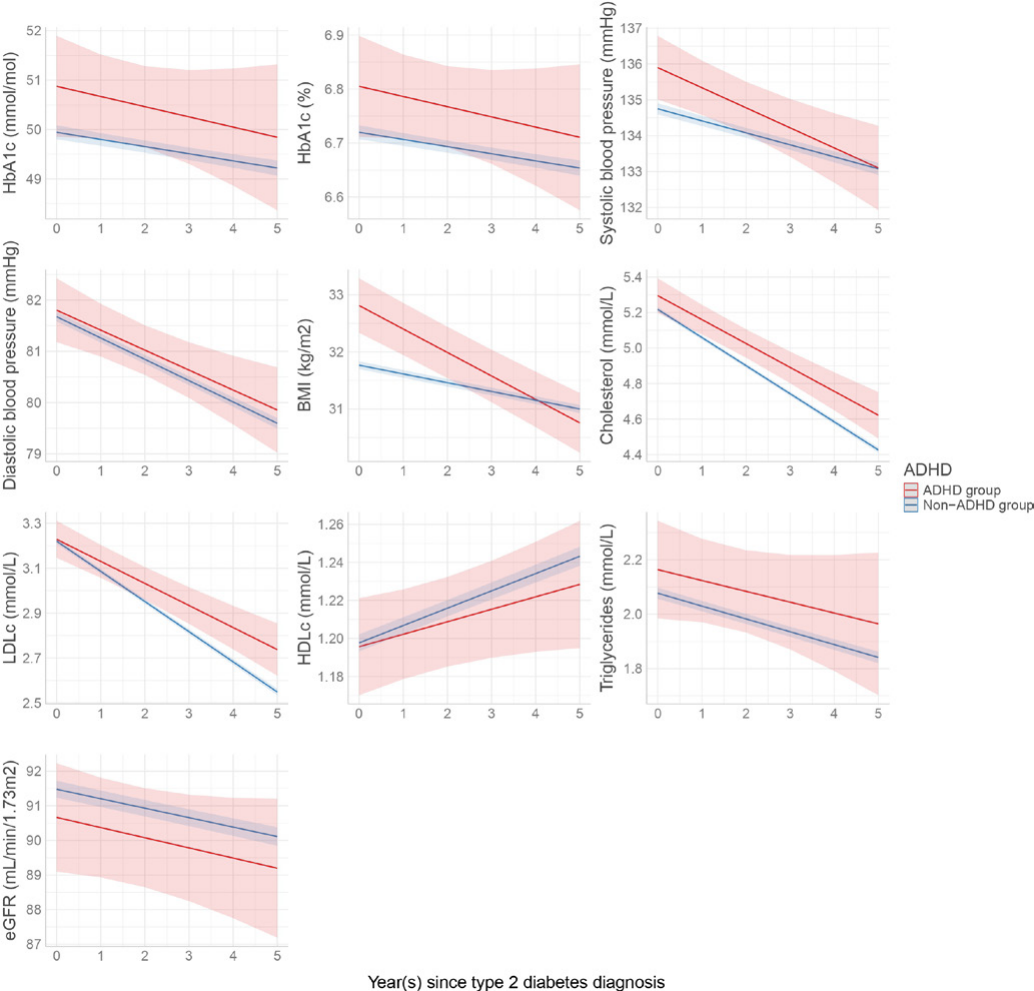
Change in clinical parameters over 5 years following the first recorded type 2 diabetes diagnosis, by ADHD group. Models were adjusted for calendar year, age and sex at type 2 diabetes diagnosis, other psychiatric disorders history and somatic disorders history. ADHD, attention-deficit/hyperactivity disorder; HbA1c, haemoglobin A1C; BMI, body mass index; LDLc, low-density lipoprotein cholesterol; HDLc, high-density lipoprotein cholesterol; eGFR, estimated glomerular filtration rate.

ADHD was associated with a significantly greater reduction in BMI in the 5 years following T2D diagnosis after adjusting for sex, age and calendar year at T2D diagnosis (β_ADHD*t_: −0.26 (95% CI −0.34 to −0.17); table 3) and psychiatric and somatic disorder history (β_ADHD*t_: −0.26 (95% CI −0.34 to −0.17); table 3, figure 1). No other associations between ADHD and changes in other clinical parameters over time were found to be statistically significant (table 3, figure 1).

**Table 3:** Association between ADHD and change in clinical parameters over 5 years following type 2 diabetes diagnosis

Clinical parameters	N		Coefficients for interaction term (95% CI)		
	ADHD	Non-ADHD	Model 1	Model 2	Model 3
HbA1c (%)	1 003	67 110	−0.01 (−0.04 to 0.03)	−0.01 (−0.04 to 0.03)	−0.01 (−0.04 to 0.03)
HbA1c (mmol/mol)			−0.06 (−0.44 to 0.31)	−0.06 (−0.43 to 0.31)	−0.06 (−0.43 to 0.31)
Systolic blood pressure (mm Hg)	891	62 489	−0.23 (−0.52 to 0.06)	−0.23 (−0.52 to 0.06)	−0.23 (−0.52 to 0.06)
Diastolic blood pressure (mm Hg)	892	62 387	0.02 (−0.19 to 0.24)	0.03 (−0.19 to 0.24)	0.03 (−0.19 to 0.24)
BMI (kg/m^2^)	799	54 672	−0.26 (−0.34 to −0.17)*	−0.26 (−0.34 to −0.17)*	−0.26 (−0.34 to −0.17)*
Total cholesterol (mmol/L)	689	50 966	0.02 (−0.01 to 0.06)	0.02 (−0.01 to 0.06)	0.02 (−0.01 to 0.06)
LDLc (mmol/L)	614	46 302	0.04 (0.01 to 0.06)	0.04 (0.01 to 0.06)	0.04 (0.01 to 0.06)
HDLc (mmol/L)	616	45 936	−0.003 (−0.01 to 0.004)	−0.003 (−0.01 to 0.004)	−0.003 (−0.01 to 0.004)
Triglycerides (mmol/L)	576	43 728	0.01 (−0.06 to 0.07)	0.01 (−0.06 to 0.07)	0.01 (−0.06 to 0.07)
eGFR (mL/min/1.73 m^2^)	851	57 736	−0.02 (−0.44 to 0.40)	−0.02 (−0.44 to 0.40)	−0.02 (−0.44 to 0.40)

Interaction term: ADHD*year(s) since first recorded type 2 diabetes diagnosis. n: Number of individuals with one baseline measurement and at least one follow-up measurement for the listed clinical parameters. Model 1: adjusted for calendar year, age and sex at type 2 diabetes diagnosis; Model 2: additionally adjusted for other psychiatric disorders’ history; Model 3 additionally adjusted for somatic disorders’ history.

* Statistically significant at a false discovery rate-corrected p-value<0.0001.

ADHD, attention-deficit/hyperactivity disorder; BMI, body mass index; eGFR, estimated glomerular filtration rate; HbA1c, haemoglobin A1C; HDLc, high-density lipoprotein cholesterol; LDLc, low-density lipoprotein cholesterol.

ADHD was not statistically significantly associated with smoking cessation or increased physical activity 2 years after T2D diagnosis. However, point estimates indicated that individuals with co-occurring T2D and ADHD were less likely to stop smoking (RR range: 0.75–0.99; table 4) and increase physical activity levels (RR range: 0.81–0.93; table 4).

**Table 4:** Association between ADHD and change in behavioural factors 2 years after type 2 diabetes diagnosis

Behavioural factors change	N (%)		Risk ratio (95% CI)*		
	ADHD	Non-ADHD	Model 1	Model 2	Model 3
Quit smoking	n=141	n=4482			
	17 (12)	724 (16)	0.75 (0.48 to 1.18)	0.99 (0.61 to 1.59)	0.99 (0.61 to 1.61)
Changed from low to high physically active	n=262	n=18 045			
	89 (34)	7 700 (43)	0.81 (0.68 to 0.96)	0.90 (0.75 to 1.07)	0.93 (0.78 to 1.11)

n: Number and proportion of individuals who had a corresponding unhealthy behavioural factor at baseline and had a corresponding behavioural factor recorded two years after baseline changed to healthy behavioural factors 2 years after baseline among individuals with ADHD or non-ADHD, respectively. For example, there are 141 individuals who were smokers at baseline with ADHD and had smoking status recorded 2 years after baseline. Among them, 17/141=12% of individuals quit smoking 2 years after baseline. Model 1: adjusted for calendar year, age and sex at type 2 diabetes diagnosis; Model 2: additionally adjusted for other psychiatric disorders’ history; Model 3: additionally adjusted for somatic disorders’ history.

* Statistically significant threshold at a false discovery rate corrected p-value<0.0001.

ADHD, attention-deficit/hyperactivity disorder.

### Stratified analyses

Stratified by sex, similar patterns of T2D diagnosis associations were observed in both females and males (online supplemental table S4). In the longitudinal analysis, a statistically significant reduction in BMI over time was observed in males with ADHD (β_ADHD*t_: −0.22 (95% CI −0.31 to −0.13); online supplemental table S5). Similar BMI longitudinal patterns were observed in females; however, these were not statistically significant (β_ADHD*t_: −0.31 (95% CI −0.47 to −0.15); online supplemental table S5). Stratified by birth cohort, significant associations between ADHD and BMI at T2D diagnosis were only observed in the younger cohort (born 1960–2000) (β_ADHD_: 1.17 (95% CI 0.67 to 1.66); online supplemental table S4). The greater reduction in BMI over time in the ADHD group remained significant for the younger cohort only (β_ADHD*t_: −0.26 (95% CI −0.36 to −0.16); online supplemental table S5).

### Sensitivity analyses

In sensitivity analyses, defining ADHD by diagnosis alone showed consistent estimates with the main analyses (online supplemental tables S6–S8). ADHD was associated with a higher baseline BMI, although no longer statistically significant, and a greater BMI reduction during follow-up (β_ADHD*t_: −0.25 (95% CI −0.34 to −0.15)). When adjusting for baseline BMI, estimates were comparable to the main analyses (online supplemental tables S9 and S10), showing significantly greater BMI reduction in those with ADHD (β_ADHD*t_: −0.26 (95% CI −0.34 to −0.17)). When considering ADHD medication use, the associations were similar between those with and without ADHD medication among individuals with ADHD (online supplemental tables S11 and S12). We observed similar, though not statistically significant, results after excluding individuals with schizophrenia, bipolar disorder and substance use disorder (online supplemental tables S13–S15), as indicated by the largely overlapping CIs with our main results. Consistent findings were also obtained when additionally adjusting for the use of antidepressants, antipsychotics and antiepileptics or second-generation antipsychotics (online supplemental tables S16–S18) for baseline BMI and subsequent BMI change. Additionally, we observed similar, though not statistically significant, results when defining ADHD status within 5 years prior to the T2D diagnosis (online supplemental tables S19–S21), aligning with our main findings. In post-hoc analysis, we additionally adjusted for glucose-lowering medication use (ATC code A10B) during follow-up to assess its role in BMI change following T2D diagnosis. Results were consistent with the main analysis, showing that ADHD remained significantly associated with greater reduction in BMI (β_ADHD*t_: −0.26 (95% CI −0.34 to −0.17)). Furthermore, after IPTW, the SMDs were reduced, and many covariates were below the conventional threshold of 0.1, indicating improved covariate balance (online supplemental table S22). The IPTW-weighted analyses yielded results consistent with the main results (online supplemental tables S23–S25) in terms of coefficient estimates and overlapping CIs, supporting the robustness of our findings. An exception was the BMI reduction post-T2D diagnosis, which was substantially reduced and not significant (β_ADHD*t_: −0.05 (95% CI −0.17 to 0.07)).

## Discussion

In this first longitudinal cohort study of individuals with T2D and co-occurring ADHD, we observed modest differences in cardiometabolic risk profiles compared with those without ADHD. At first recorded T2D, individuals with ADHD had a higher BMI, elevated triglycerides and were more often smokers. The association between ADHD and BMI attenuated but remained statistically significant after adjusting for psychiatric and somatic comorbidities. Over the 5 years following T2D diagnosis, individuals with ADHD showed a greater reduction in BMI compared with those without ADHD, independent of baseline BMI and glucose-lowering medication use. Despite modest overall cardiometabolic differences between individuals with and without ADHD, incorporating ADHD status into preventive diabetes care may help clinicians identify a younger, more vulnerable subgroup who could benefit from targeted cardiometabolic risk factor management. 

### Interpretation

The mean BMI for both the ADHD and non-ADHD groups at T2D diagnosis was over 30, classifying them as obese per WHO criteria. The ADHD group had a mean BMI nearly two units higher, and this association remained significant after adjusting for psychiatric and somatic disorder history, with the ADHD group still showing almost one unit higher BMI. This finding is consistent with existing evidence linking ADHD to higher BMI and obesity,^7 8 11 13^ and suggests that core ADHD symptoms, such as impulsivity and disorganisation, may lead to unhealthy lifestyle practices and addictive eating behaviours (eg, binge eating).^44^ The association between ADHD and higher BMI may also be due to common genetic vulnerabilities influencing both conditions. For example, a genome-wide association meta-analysis revealed significant genetic overlap between ADHD and obesity-related phenotypes.^45^ Triglyceride levels exceeded the desired threshold (<1.7 mmol/L) in both groups at T2D diagnosis, with the ADHD group demonstrating slightly higher mean triglyceride. The finding contradicts a recent study showing no statistically significant association,^26^ but this is potentially explained by differences in ADHD definitions, sampling methods, sample size and triglyceride measurement time. A recent narrative review reported that several psychotropic medications, including second-generation antipsychotics, antidepressants and antiepileptics, are associated with weight gain.^42^ Our sensitivity analyses, excluding individuals with severe psychiatric comorbidities and adjusting for psychotropic medication use, yielded similar results, suggesting that ADHD may independently contribute to higher BMI at T2D diagnosis. Our observed higher prevalence of smoking in the ADHD group is consistent with existing studies in the general ADHD population.^8 9 14^ However, the association between ADHD and triglycerides, as well as smoking, was attenuated and no longer significant after adjusting for other psychiatric and somatic disorders, suggesting that the difference in triglycerides and smoking may not be primarily explained by ADHD.

While the ADHD group in our study displayed a different profile in other clinical parameters (HbA1c, blood pressure, total cholesterol, HDLc, LDLc and eGFR) and physical activity levels compared with the non-ADHD group at T2D diagnosis, these differences were minor and not statistically significant. Both groups exceed the target management thresholds for mean blood pressure, total cholesterol and LDLc. Existing evidence on these clinical parameters and physical activity in relation to ADHD remains inconsistent,^11 12 15 16 20 26^ while research among individuals with co-occurring T2D and ADHD remains even scarcer, highlighting the need for future research. ADHD has been consistently associated with higher HbA1c in individuals with T1D, which seems to be mainly attributed to the impaired executive functions caused by ADHD.^24^ However, more research is needed to evaluate whether individuals with co-occurring T2D and ADHD also face challenges in achieving appropriate glycaemic control.

Following individuals over the years after T2D diagnosis, both the ADHD and non-ADHD groups showed improvements, to a similar degree, in HbA1c, blood pressure and lipid profiles. One exception was that individuals with co-occurring T2D and ADHD tended to show greater reductions in BMI, even after adjusting for baseline BMI levels and glucose-lowering medication use. To our knowledge, there have been no prior studies investigating changes in BMI related to ADHD in individuals with T2D. Studies have reported that ADHD medication is linked with weight loss in early treatment stages,^46 47^ which could be a possible explanation of findings. However, our sensitivity analysis, excluding those using ADHD medication, yielded similar associations, suggesting that the finding is unlikely to be explained by ADHD medication alone. Another plausible explanation for the greater BMI reduction is that endocrinologists may be more likely to prescribe weight loss treatments to individuals with ADHD, given their poorer BMI profile at diabetes diagnosis. While glucagon-like peptide-1 receptor agonists (GLP-1 RAs) are effective for weight loss,^48^ their impact on our findings is likely minimal. Their use in Sweden was low throughout most of the study period (1996–January 2023),^49 50^ with increased uptake only from 2022 onwards, making differential exposure by ADHD status unlikely to have influenced results. We did observe, however, that the reduction in BMI following T2D diagnosis substantially attenuated in IPTW analyses, suggesting that this association is likely driven by differences in baseline characteristics and comorbidities rather than an independent effect of ADHD. Further research is needed to elucidate the underlying mechanisms and disentangle potential sources of confounding. We found limited evidence that ADHD was associated with smoking cessation or increased physical activity, likely due to limited statistical power. Further research is needed to clarify the impact of ADHD on behavioural changes in individuals with T2D.

Stratified analyses revealed generally comparable associations of ADHD and cardiometabolic risk profile between females and males. Stratification by birth cohort suggested that the higher BMI at T2D diagnosis and greater subsequent BMI reduction among individuals with ADHD were primarily observed in younger birth cohorts, in whom ADHD diagnosis is likely more completely ascertained, as ADHD is underdiagnosed among older generations.^51^ This pattern indicates that the main findings are unlikely to be explained solely by age imbalance or secular changes in ADHD diagnosis. However, ADHD ascertainment in older birth cohorts is likely incomplete, and the smaller number of older individuals with ADHD limits interpretation of null findings in this subgroup. Future studies are needed to explore the birth cohort effects with greater statistical power and without solely relying on clinical diagnoses of ADHD in the elderly.

### Strengths and limitations

There are several strengths to highlight in this nationwide register study. First, the Swedish National Diabetes Register contains detailed, continuously collected data on clinical parameters and behavioural factors of individuals with diabetes, covering most individuals with T2D visiting healthcare facilities in Sweden (87% coverage in 2019).^49^ Furthermore, data were collected prospectively, minimising recall bias. The use of ICD codes from reliable patient registers with high national coverage helped mitigate misclassification biases of psychiatric and somatic disorders.^32^

Limitations of our study are also important to consider. First, register data only captures individuals who visited healthcare facilities, potentially overrepresenting those with more severe conditions. While it is also possible that individuals with more severe ADHD symptoms may be less likely to undergo clinical measurements and may have poorer metabolic profiles, the underrepresentation of such individuals might contribute to more conservative estimates in our study. Second, the Prescribed Drug Register was established in 2005, potentially leading to incomplete exclusion of individuals with T2D medication history before T2D diagnosis. However, this is unlikely to impact our findings, as only 8.7% of the sample was diagnosed with T2D before 2005, and results remained consistent when these individuals were excluded (online supplemental table S16–S18). Third, register coverage, diagnostic practices and awareness of ADHD have improved over the past decades,^52^ reflected in increased ADHD diagnoses in the younger cohort and a subsequent overrepresentation of younger individuals within the ADHD group (online supplemental table S26). This might result in power constraints and potential misclassification of ADHD in the older cohort; however, our stratified analyses across cohorts showed similar estimates to the main findings. Fourth, given the observational nature of this study, residual confounding cannot be excluded. However, our sensitivity analyses using IPTW and stratification by birth cohort and sex generally suggested robustness in the main results. For the longitudinal analyses, the greater BMI reduction following T2D diagnosis among individuals with ADHD persisted after adjustment for obesity and baseline BMI but was attenuated after IPTW adjustment accounting for age, sex, birth year and somatic and psychiatric comorbidities. These findings should therefore be interpreted with caution, as the observed BMI changes may partly reflect differences in baseline characteristics, metabolic risk profiles and potential for weight loss between groups. Lastly, this study aimed to outline cardiometabolic risk profiles in individuals with and without ADHD. Future research should investigate whether greater BMI reduction over time relates to differences in medication use for diabetes, hypertension and high cholesterol, which was beyond this study’s scope.

## Conclusions

The findings of this study suggest that individuals with co-occurring T2D and ADHD present with a modestly worse cardiometabolic profile at T2D diagnosis, with a higher BMI, triglycerides and more smoking behaviours, compared with those without ADHD. On the other hand, individuals with T2D and ADHD achieved greater BMI reduction following the T2D diagnosis. These findings indicate that individuals with co-occurring ADHD and T2D may warrant closer cardiometabolic assessment, though replication in independent cohorts and investigation of underlying mechanisms are needed. While cardiometabolic profiles were overall similar between groups, if replicated, these findings suggest that incorporating ADHD awareness into pre-diabetes and diabetes care may facilitate earlier clinical attention directed toward weight management, blood lipid control and smoking cessation. Such an approach would be particularly relevant for identifying a younger and more vulnerable subgroup who may present with an elevated initial cardiometabolic risk profile at T2D diagnosis.

## Supplementary material

10.1136/bmjopen-2025-113372 online supplemental file 1

## Data Availability

Data may be obtained from a third party and are not publicly available.
