# Supplementary material for: ADHD and cardiometabolic risk profile in adults with type 2 diabetes: a longitudinal register-based study
Source: BMJ Open. 2026 Jul 3;16(7):e113372. doi: 10.1136/bmjopen-2025-113372 (PMC13343064; doi:10.1136/bmjopen-2025-113372)
Supplement: online supplemental file 1 [file bmjopen-16-7-s001.docx]

**Supplementary data**

sMethod. Description of Swedish registers 3

Figure S1. Flowchart of study population selection 4

Figure S2. Sketch plot of study design 5

Table S1. Description of cardiometabolic risk profile in Swedish National Diabetes Register 6

Table S2. Anatomical Therapeutic Chemical (ATC) codes and International Classification of Diseases (ICD) Revisions codes used to define historic psychiatric and somatic disorders 7

Table S3. Median number of clinical parameters taken by individual during follow-up, by ADHD group 8

Figure S3. Median years since type 2 diabetes diagnosis and number of individuals for n^th^ (i.e., 1^st^, 2^nd^, 3^rd^…) measurement of each clinical parameters during follow-up, by ADHD group 9

Table S4. Association between ADHD and cardiometabolic risk profile at first recorded type 2 diabetes diagnosis (baseline), stratified by sex and birth cohort 10

Table S5. Association between ADHD and change in clinical parameters over five years following first recorded type 2 diabetes diagnosis, stratified by sex and birth cohort 12

Table S6. Association between ADHD and cardiometabolic risk profile at first recorded type 2 diabetes diagnosis (baseline), where ADHD was defined only by diagnosis at Patient Register using ICD code 14

Table S7. Association between ADHD and change in clinical parameters over five years following first recorded type 2 diabetes diagnosis, where ADHD was defined only by diagnosis at Patient Register using ICD code 15

Table S8. Association between ADHD and change in behavioural factors two years following first recorded type 2 diabetes diagnosis, where ADHD was defined only by diagnosis at Patient Register using ICD code 16

Table S9. Association between ADHD and clinical parameters at first recorded type 2 diabetes diagnosis (baseline), adjusting for baseline BMI 17

Table S10. Association between ADHD and change in clinical parameters over five years following first recorded type 2 diabetes diagnosis, additionally adjusting for baseline BMI 18

Table S11. Association between ADHD and change in clinical parameters over five years following first recorded type 2 diabetes diagnosis, where ADHD examined as a three-category variable 19

Table S12. Association between ADHD and change in behavioural factors two years after first recorded type 2 diabetes diagnosis, where ADHD examined as a three-category variable 20

Table S13. Association between ADHD and cardiometabolic risk profile at type 2 diabetes diagnosis, when excluded study population with history of schizophrenia or bipolar disorder or substance use disorder 21

Table S14. Association between ADHD and change in clinical parameters over five years following type 2 diabetes diagnosis, when excluded study population with history of schizophrenia or bipolar disorder or substance use disorder 22

Table S15. Association between ADHD and change in behavioural factors two years after type 2 diabetes diagnosis, when excluded study population with history of schizophrenia or bipolar disorder or substance use disorder 23

Table S16. Association between ADHD and cardiometabolic risk profile at type 2 diabetes diagnosis, when further adjusting for psychotropic medication use within 3 months before type 2 diabetes diagnosis 24

Table S17. Association between ADHD and change in clinical parameters over five years following type 2 diabetes diagnosis, when further adjusting for psychotropic medication use within 3 months before type 2 diabetes diagnosis 25

Table S18. Association between ADHD and change in behavioural factors two years after type 2 diabetes diagnosis, when further adjusting for psychotropic medication use within 3 months before type 2 diabetes diagnosis 26

Table S19. Association between ADHD and cardiometabolic risk profile at type 2 diabetes diagnosis, when examining ADHD status within 5 years before type 2 diabetes diagnosis 27

Table S20. Association between ADHD and change in clinical parameters over five years following type 2 diabetes diagnosis, when examining ADHD status within 5 years before type 2 diabetes diagnosis 28

Table S21. Association between ADHD and change in behavioural factors two years after type 2 diabetes diagnosis, when examining ADHD status within 5 years before type 2 diabetes diagnosis 29

Table S22. Comparison of standardized mean differences before and after applying inverse probability of treatment weighting 30

Table S23. Association between ADHD and cardiometabolic risk profile at first recorded type 2 diabetes diagnosis (baseline), after applying inverse probability of treatment weighting 31

Table S24. Association between ADHD and change in clinical parameters over five years following first recorded type 2 diabetes diagnosis, after applying inverse probability of treatment weighting 32

Table S25. Association between ADHD and change in behavioural factors two years following first recorded type 2 diabetes diagnosis, after applying inverse probability of treatment weighting 33

Table S26. Characteristics of individuals with and without ADHD in the cohort of individuals with type 2 diabetes at first recorded type 2 diabetes diagnosis (baseline), stratified by birth cohort and sex 34

# sMethod. Description of Swedish registers

We used data from the following Swedish nationwide registers, linked via unique personal identification numbers:^1^ the Total Population Register, covering demographic information since 1968 and migration information since 1969;^2^ the National Patient Register includes data on inpatient care since 1964 and outpatient care since 2001, with psychiatric diagnoses available from 1973. Complete inpatient coverage began in 1987, and all diagnoses are recorded according to the International Classification of Diseases (ICD);^3^ the Prescribed Drug Register, covering all medication dispensations since 2005, using the Anatomic Therapeutic Chemical (ATC) code^4^ and the Swedish National Diabetes Register, established in 1996, covering 100% of specialist clinics and 90% of primary health care centres (responsible for treatment of majority individuals with type 2 diabetes) in Sweden^5^.

# Figure S1. Flowchart of study population selection


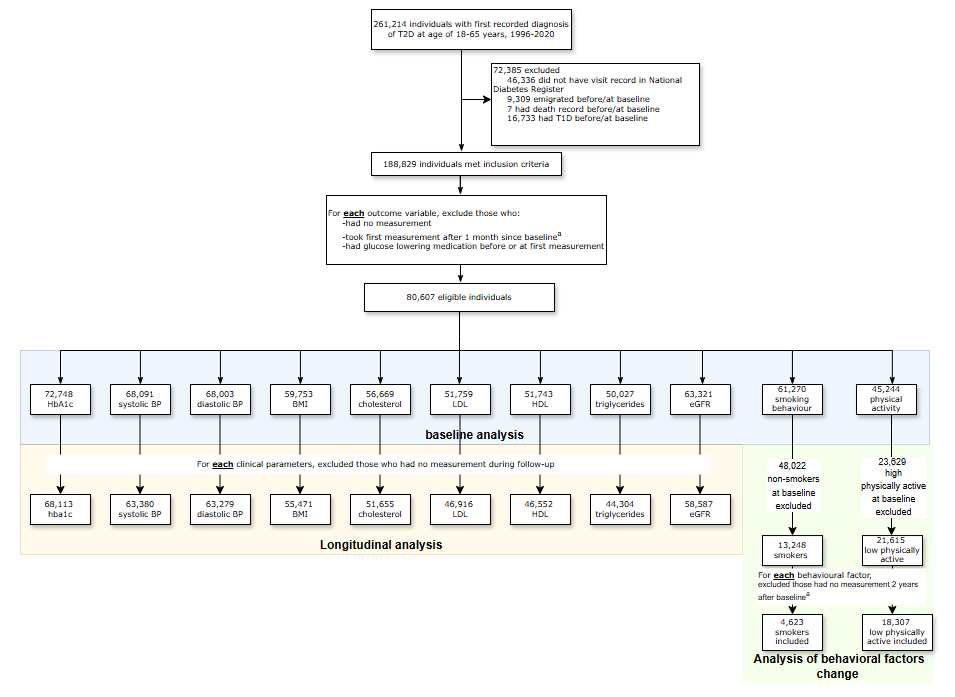


Abbreviation: T2D: type 2 diabetes, T1D: type 1 diabetes, HbA1c: haemoglobin A1C, BP: blood pressure, BMI: body mass index, LDLc: low-density lipoprotein cholesterol, HDLc: high-density lipoprotein cholesterol, eGFR: estimated glomerular filtration rate

^a^ Baseline refers to first recorded type 2 diabetes diagnosis

# Figure S2. Sketch plot of study design


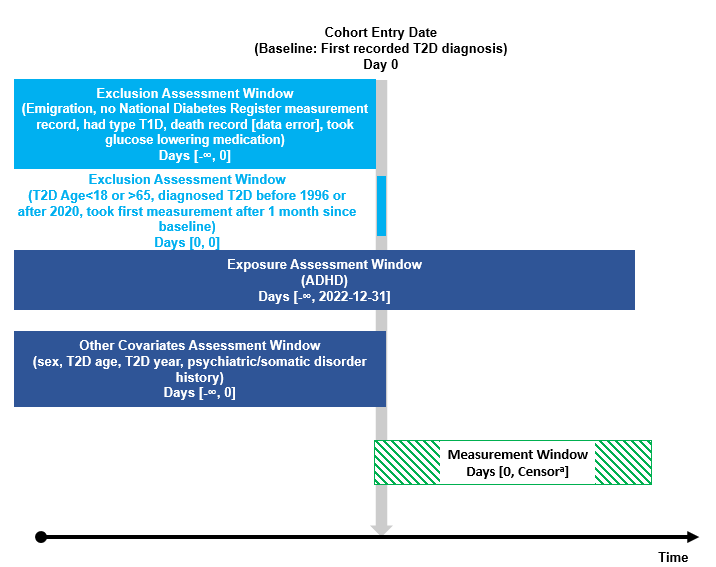


Abbreviation: ADHD: attention-deficit/hyperactivity disorder, T2D: type 2 diabetes, T1D: type 1 diabetes

^a^ Earliest of: 5 years of follow-up, end of the study period (2023-01-30)

# Table S1. Description of cardiometabolic risk profile in Swedish National Diabetes Register

|  | **Description** | **Value** |
| --- | --- | --- |
| **Clinical parameters** |  |  |
| HbA1c (mmol/mol) | If value is below 20, value is calculated from Diabetes Control and Complications Trial (DCTT) unit to International Federation of Clinical Chemistry (IFCC) unit | integer |
| Systolic blood pressure (mmHg) | Collected after sitting for five minutes of rest | integer |
| Diastolic blood pressure (mmHg) | Collected after sitting for five minutes of rest | integer |
| BMI (kg/m^2^) | Calculated if input parameters are reported during clinical visit | decimal number, 1 decimal place |
| Total cholesterol (mmol/L) | Collected through laboratory test, indicating total cholesterol level in the blood | decimal number, 1 decimal place |
| LDLc (mmol/L) | Calculated according to the Friedewald formula if input parameters (i.e., total cholesterol, HDLc and triglycerides) are reported and LDL is not reported | decimal number, 1 decimal place |
| HDLc (mmol/L) | Collected through laboratory test, indicating high-density lipoprotein cholesterol level in the blood | decimal number, 1 decimal place |
| Triglycerides (mmol/L) | Collected through laboratory test, indicating triglycerides level in the blood | decimal number, 1 decimal place |
| eGFR (mL/min/1.73m^2^) | Calculated with Modification of Diet in Renal Disease (MDRD) formula if creatinine is reported | decimal number, 2 decimal places |
| **Behavioural factors** |  |  |
| Smoking | Collected through self-report. One or more cigarettes per day or use smoking pipe or quit less than three months | 0=no  1=yes |
| Physical activity^a^ | Collected through self-report. 30 min walk or equivalent. Total physical activity of at least 30 minutes, where sessions of 10 minutes can be added up. Any activity that can cause light pulse-raising. | 1=never  2=once a week  3=1-2 times a week  4=3-5 times a week  5=daily |

Abbreviation: HbA1c: haemoglobin A1C, BMI: body mass index, LDLc: low-density lipoprotein cholesterol, HDLc: high-density lipoprotein cholesterol, eGFR: estimated glomerular filtration rate

^a^ In the analyses, for physical activity, we combined level 1 (no physical activity), level 2 (once a week) and level 3 (1-2 times a week) as “low physically active”, level 4 (3-5 times a week) and level 5 (daily physical activity) as “high physically active”.

# Table S2. Anatomical Therapeutic Chemical (ATC) codes and International Classification of Diseases (ICD) Revisions codes used to define historic psychiatric and somatic disorders

|  | **ICD codes from National Patient Register** | | | **ATC codes** |
| --- | --- | --- | --- | --- |
|  | **ICD-8 Codes** | **ICD-9 Codes** | **ICD-10 Codes** |  |
| Obesity | 277 | 278.A, 278.B | E65, E66 | - |
| Cardiovascular diseases | 39-43, 440, 444, 445, 450-453, 458 | 390-430, 440, 444, 445 | I0-I6, I70, I730, I74, I75 | - |
| Hyperlipidaemia | 279 | 272 | E78 | C10 |
| Sleep disorders | 306.40 | 307E, 780F | G47 | - |
| Anxiety disorders | 300.0 | 300.00,300.02 | F40-F41 | - |
| Autism spectrum disorder | - | 299 | F84 | - |
| Bipolar disorder | 296.1, 296.3, 296.8 | 296A/ C/D/E/W | F30-F31 | - |
| Conduct disorder | - | 312 | F91 | - |
| Major depressive disorder | 296.2, 298.0,300.4 | 296B,300E | F32-33 | - |
| Eating disorders | 306.5x | 307.5 | F50 | - |
| Intellectual disability | 310-315 | 317-319 | F7 | - |
| Personality disorders | 301 | 301 | F60 | - |
| Schizophrenia and psychotic disorders | 295 except 295.7 | 295A-E/G/W/X | F2 | - |
| Substance use disorder | 291, 303, 304 | 291, 292, 304, 305A, 305X | F10-16, F18-F19 | - |

#

# Table S3. Median number of clinical parameters taken by individual during follow-up, by ADHD group

|  | **Number of measurements (median [IQR])** | |
| --- | --- | --- |
|  | **Non-ADHD** | **ADHD** |
| HbA1c (%) | 7[5,10] | 7[4,10] |
| HbA1c (mmol/mol) |  |  |
| Systolic blood pressure (mmHg) | 6[4,10] | 6[4,10] |
| Diastolic blood pressure (mmHg) | 6[4,10] | 6[4,10] |
| BMI (kg/m^2^) | 6[4,8] | 6[4,9] |
| Total cholesterol (mmol/L) | 5[3,7] | 5[3,7] |
| LDLc (mmol/L) | 5[3,7] | 5[3,7] |
| HDLc (mmol/L) | 5[3,7] | 5[3,7] |
| Triglycerides (mmol/L) | 5[3,7] | 5[3,7] |
| eGFR (mL/min/1.73m^2^) | 6[4,8] | 5[3,8] |

Abbreviation: ADHD: attention-deficit/hyperactivity disorder

# Figure S3. Median years since type 2 diabetes diagnosis and number of individuals for n^th^ (i.e., 1^st^, 2^nd^, 3^rd^…) measurement of each clinical parameters during follow-up, by ADHD group


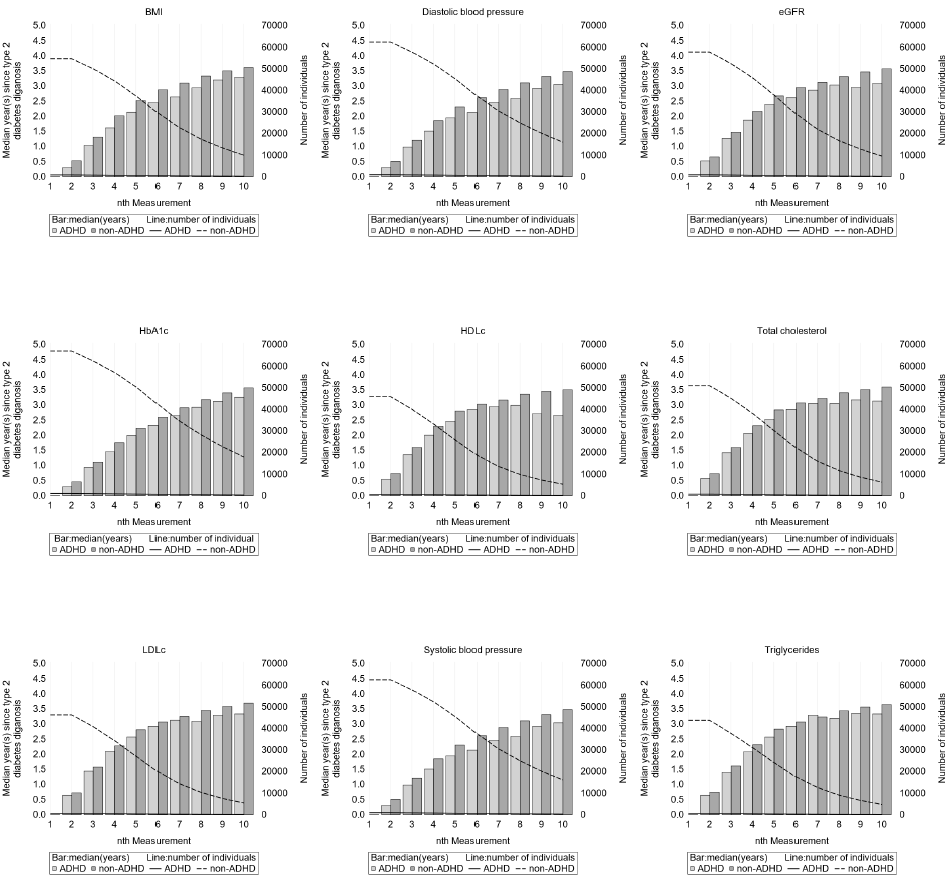


Abbreviation: ADHD: attention-deficit/hyperactivity disorder, HbA1c: haemoglobin A1C, BMI: body mass index, LDLc: low-density lipoprotein cholesterol, HDLc: high-density lipoprotein cholesterol, eGFR: estimated glomerular filtration rate

Interpretation: For example, 50 057 individuals in non-ADHD group and 711 individuals in ADHD group received their third BMI measurements at (median) year 1.3 and 1.0 after type 2 diabetes diagnosis, respectively.

# Table S4. Association between ADHD and cardiometabolic risk profile at first recorded type 2 diabetes diagnosis (baseline), stratified by sex and birth cohort

| **Clinical parameters** | **n** | | **Mean (SD)** | | **Beta coefficients**  **(95%CI)** |
| --- | --- | --- | --- | --- | --- |
|  | **ADHD** | **Non-ADHD** | **ADHD** | **Non-ADHD** |  |
| **By sex - Male** |  |  |  |  |  |
| HbA1c (%) | 657 | 42 054 | 7.40 (1.89) | 7.09 (1.68) | -0.04 (-0.17,0.10) |
| HbA1c (mmol/mol) |  |  | 57.42 (20.70) | 54.04 (18.38) | -0.42 (-1.90,1.07) |
| Systolic blood pressure (mmHg) | 597 | 39 473 | 134.36 (15.35) | 136.89 (16.56) | 1.26 (-0.15,2.67) |
| Diastolic blood pressure (mmHg) | 598 | 39 436 | 84.89 (10.49) | 83.23 (10.19) | 0.93 (0.06,1.81) |
| BMI (kg/m^2^) | 523 | 34 597 | 34.63 (6.81) | 31.37 (5.49) | 0.76 (0.29,1.23) |
| Total cholesterol (mmol/L) | 495 | 33 342 | 5.40 (1.42) | 5.24 (1.20) | 0.06 (-0.05,0.17) |
| LDLc (mmol/L) | 430 | 30 281 | 3.32 (1.08) | 3.25 (1.02) | -0.001 (-0.10,0.10) |
| HDLc (mmol/L) | 444 | 30 439 | 1.03 (0.31) | 1.13 (0.33) | -0.01 (-0.04,0.02) |
| Triglycerides (mmol/L) | 418 | 29 418 | 2.98 (2.68) | 2.30 (2.04) | 0.19 (-0.02,0.40) |
| eGFR (mL/min/1.73m^2^) | 567 | 36 826 | 102.26 (25.07) | 92.64 (21.70) | -0.05 (-1.84,1.75) |
| **By sex - Female** |  |  |  |  |  |
| HbA1c (%) | 426 | 29 611 | 6.96 (1.52) | 6.82 (1.41) | 0.06 (-0.08,0.21) |
| HbA1c(mmol/mol) |  |  | 52.55 (16.59) | 50.98 (15.45) | 0.68 (-0.89,2.24) |
| Systolic blood pressure (mmHg) | 377 | 27 644 | 128.77 (14.73) | 135.01 (16.81) | 0.47 (-1.28,2.21) |
| Diastolic blood pressure (mmHg) | 378 | 27 591 | 80.48 (9.99) | 81.10 (9.82) | -0.56 (-1.61,0.50) |
| BMI (kg/m^2^) | 346 | 24 287 | 36.65 (7.71) | 32.61 (6.47) | 1.15 (0.48,1.83) |
| Total cholesterol (mmol/L) | 288 | 22 544 | 5.44 (1.24) | 5.45 (1.15) | 0.07 (-0.07,0.21) |
| LDLc (mmol/L) | 268 | 20 780 | 3.44 (1.09) | 3.40 (1.01) | 0.07 (-0.05,0.20) |
| HDLc (mmol/L) | 264 | 20 596 | 1.16 (0.34) | 1.30 (0.38) | -0.01 (-0.06,0.04) |
| Triglycerides (mmol/L) | 250 | 19 941 | 2.29 (1.59) | 1.94 (1.28) | 0.02 (-0.14,0.19) |
| eGFR (mL/min/1.73m^2^) | 371 | 25 557 | 96.95 (25.82) | 89.70 (23.29) | -1.65 (-3.98,0.68) |
| **By birth cohort - 1930-1959** |  |  |  |  |  |
| HbA1c (%) | 198 | 44789 | 6.96 (1.56) | 6.83 (1.41) | 0.06 (-0.14,0.26) |
| HbA1c (mmol/mol) |  |  | 52.60 (17.04) | 51.18 (15.45) | 0.66 (-1.51,2.84) |
| Systolic blood pressure (mmHg) | 186 | 42590 | 133.54 (15.05) | 137.92 (16.48) | -1.90 (-4.29,0.50) |
| Diastolic blood pressure (mmHg) | 187 | 42535 | 81.36 (9.69) | 81.87 (9.68) | -0.67 (-2.07,0.74) |
| BMI (kg/m^2^) | 168 | 37 421 | 32.06 (5.93) | 31.03 (5.40) | 0.11 (-0.67,0.89) |
| Total cholesterol (mmol/L) | 145 | 35 391 | 5.46 (1.21) | 5.33 (1.16) | 0.10 (-0.09,0.28) |
| LDLc (mmol/L) | 136 | 32 040 | 3.44 (1.06) | 3.27 (1.02) | 0.15 (-0.01,0.32) |
| HDLc (mmol/L) | 130 | 32 097 | 1.22 (0.37) | 1.24 (0.37) | 0.01 (-0.05,0.07) |
| Triglycerides (mmol/L) | 124 | 31 550 | 2.31 (1.79) | 2.03 (1.50) | 0.07 (-0.20,0.33) |
| eGFR (mL/min/1.73m^2^) | 179 | 38 999 | 87.91 (19.98) | 86.58 (19.83) | -0.38 (-3.27,2.52) |
| **By birth cohort - 1960-2002** |  |  |  |  |  |
| HbA1c (%) | 885 | 26 876 | 7.29 (1.81) | 7.22 (1.80) | 0.03 (-0.10,0.16) |
| HbA1c (mmol/mol) |  |  | 56.16 (19.75) | 55.44 (19.72) | 0.31 (-1.10,1.71) |
| Systolic blood pressure (mmHg) | 788 | 24 527 | 131.88 (15.41) | 132.99 (16.58) | 1.38 (0.14,2.61) |
| Diastolic blood pressure (mmHg) | 789 | 24 492 | 83.62 (10.66) | 83.19 (10.72) | 0.93 (0.12,1.74) |
| BMI (kg/m^2^) | 701 | 21 463 | 36.24 (7.30) | 33.37 (6.55) | 1.17 (0.67,1.66)^*^ |
| Total cholesterol (mmol/L) | 638 | 20 495 | 5.41 (1.39) | 5.33 (1.23) | 0.08 (-0.03,0.18) |
| LDLc (mmol/L) | 562 | 19 021 | 3.34 (1.09) | 3.36 (1.01) | -0.001 (-0.09,0.09) |
| HDLc (mmol/L) | 578 | 18 938 | 1.05 (0.31) | 1.11 (0.32) | -0.02 (-0.05,0.01) |
| Triglycerides (mmol/L) | 544 | 17 809 | 2.82 (2.46) | 2.37 (2.18) | 0.16 (-0.04,0.36) |
| eGFR (mL/min/1.73m^2^) | 759 | 23 384 | 103.05 (25.80) | 99.54 (24.07) | -0.80 (-2.59,0.98) |
| **Behavioural factors** | **n (%)** | | **Risk Ratio (95%CI)^*^** | | |
|  | **ADHD** | **Non-ADHD** |  | | |
| **By sex- Male** |  |  |  | | |
| Smoking | N=503 | N=35 515 |  | | |
|  | 174 (35) | 7 522 (21) | 1.08 (0.95,1.23) | | |
| Low physically active | N=379 | N=26 083 |  | | |
|  | 208 (55) | 12 648 (48) | 0.98 (0.89,1.08) | | |
| **By sex- Female** |  |  |  | | |
| Smoking | N=347 | N=24 905 |  | | |
|  | 133 (38) | 5 419 (22) | 1.24 (1.06,1.44) | | |
| Low physically active | N=261 | N=18 521 |  | | |
|  | 143 (55) | 8 616 (47) | 1.00 (0.88,1.12) | | |
| **By birth cohort- 1930-1959** |  |  |  | | |
| Smoking | N=166 | N=38 862 |  | | |
|  | 56 (34) | 8 194 (21) | 1.08 (0.86,1.35) | | |
| Low physically active | N=124 | N=27 606 |  | | |
|  | 57 (46) | 12 690 (46) | 0.87 (0.72,1.06) | | |
| **By birth cohort- 1960-2002** |  |  |  | | |
| Smoking | N=684 | N=21 558 |  | | |
|  | 251 (37) | 4 747 (22) | 1.19 (1.06,1.33) | | |
| Low physically active | N=516 | N=16 998 |  | | |
|  | 294 (57) | 8 574 (50) | 1.04 (0.96,1.13) | | |

Abbreviation: ADHD: attention-deficit/hyperactivity disorder, HbA1c: haemoglobin A1C, BMI: body mass index, LDLc: low-density lipoprotein cholesterol, HDLc: high-density lipoprotein cholesterol, eGFR: estimated glomerular filtration rate

Target management levels for clinical parameters in individuals with type 2 diabetes : HbA1c: <7% (53 mmol/mol); Diastolic Blood Pressure: <80 mmHg; Systolic Blood Pressure: <130 mmHg; BMI: <25 kg/m²; Total Cholesterol: <4 mmol/L; LDLc: <2.6 mmol/L; HDLc: >1.0 mmol/L; Triglycerides: <1.7 mmol/L; eGFR: ≥90 mL/min/1.73 m²

n: For clinical parameters, refers to number of individuals with corresponding measurements; for behavioural factors, refers to number and proportion of individuals who are smokers or have low physical activity level among those whose smoking status or physical activity levels were measured for ADHD group and non-ADHD group at type 2 diabetes diagnosis. Model adjusted for year at baseline, age at baseline, sex, other psychiatric disorders history, somatic disorders history

^*^ Statistically significant at a false discovery rate corrected p value of <0.0001

# Table S5. Association between ADHD and change in clinical parameters over five years following first recorded type 2 diabetes diagnosis, stratified by sex and birth cohort

| **Clinical parameters** | **n** | | **Coefficients for interaction term (95%CI)** |
| --- | --- | --- | --- |
|  | **ADHD** | **Non-ADHD** |  |
| **By sex - Male** |  |  |  |
| HbA1c (%) | 615 | 39 456 | -0.03 (-0.07,0.02) |
| HbA1c (mmol/mol) |  |  | -0.30 (-0.79,0.20) |
| Systolic blood pressure (mmHg) | 553 | 36 871 | -0.38 (-0.74,-0.02) |
| Diastolic blood pressure (mmHg) | 554 | 36 822 | -0.08 (-0.35,0.19) |
| BMI (kg/m^2^) | 485 | 32 211 | -0.22 (-0.31,-0.13)^*^ |
| Total cholesterol (mmol/L) | 438 | 30 420 | 0.01 (-0.03,0.05) |
| LDLc (mmol/L) | 381 | 27 469 | 0.03 (-0.01,0.06) |
| HDLc (mmol/L) | 388 | 27 394 | -0.004 (-0.01,0.01) |
| Triglycerides (mmol/L) | 361 | 26 062 | -0.02 (-0.11,0.08) |
| eGFR (mL/min/1.73m^2^) | 519 | 34 090 | -0.12 (-0.68,0.45) |
| **By sex - Female** |  |  |  |
| HbA1c (%) | 388 | 27 654 | 0.03 (-0.02,0.08) |
| HbA1c (mmol/mol) |  |  | 0.30 (-0.24,0.85) |
| Systolic blood pressure (mmHg) | 338 | 25 618 | 0.03 (-0.46,0.52) |
| Diastolic blood pressure (mmHg) | 338 | 25 565 | 0.21 (-0.14,0.56) |
| BMI (kg/m^2^) | 314 | 22 461 | -0.31 (-0.47,-0.15) |
| Total cholesterol (mmol/L) | 251 | 20 546 | 0.05 (-0.002,0.11) |
| LDLc (mmol/L) | 233 | 18 833 | 0.05 (0.01,0.10) |
| HDLc (mmol/L) | 228 | 18 542 | 0.001 (-0.01,0.01) |
| Triglycerides (mmol/L) | 215 | 17 666 | 0.05 (-0.02,0.12) |
| eGFR (mL/min/1.73m^2^) | 332 | 23 646 | 0.13 (-0.47,0.74) |
| **By birth cohort- 1930-1959** |  |  |  |
| HbA1c (%) | 185 | 41 862 | 0.03 (-0.03,0.08) |
| HbA1c (mmol/mol) |  |  | 0.29 (-0.31,0.90) |
| Systolic blood pressure (mmHg) | 170 | 39 620 | -0.06 (-0.71,0.59) |
| Diastolic blood pressure (mmHg) | 171 | 39 555 | 0.06 (-0.44,0.57) |
| BMI (kg/m^2^) | 155 | 34 788 | -0.02 (-0.20,0.16) |
| Total cholesterol (mmol/L) | 133 | 32 370 | -0.04 (-0.11,0.03) |
| LDLc (mmol/L) | 127 | 29 031 | -0.02 (-0.08,0.04) |
| HDLc (mmol/L) | 118 | 28 892 | -0.01 (-0.02,0.01) |
| Triglycerides (mmol/L) | 112 | 28 052 | -0.09 (-0.17,-0.004) |
| eGFR (mL/min/1.73m^2^) | 165 | 36 202 | 0.38 (-0.46,1.22) |
| **By birth cohort- 1960-2002** |  |  |  |
| HbA1c (%) | 818 | 25 248 | -0.002 (-0.04,0.04) |
| HbA1c (mmol/mol) |  |  | -0.02 (-0.46,0.43) |
| Systolic blood pressure (mmHg) | 721 | 22 869 | -0.37 (-0.70,-0.04) |
| Diastolic blood pressure (mmHg) | 721 | 22 832 | -0.12 (-0.36,0.11) |
| BMI (kg/m^2^) | 644 | 19 884 | -0.26 (-0.36,-0.16)^*^ |
| Total cholesterol (mmol/L) | 556 | 18 596 | 0.06 (0.02,0.09) |
| LDLc (mmol/L) | 487 | 17 271 | 0.06 (0.03,0.09) |
| HDLc (mmol/L) | 498 | 17 044 | -0.003 (-0.01,0.005) |
| Triglycerides (mmol/L) | 464 | 15 676 | 0.05 (-0.03,0.13) |
| eGFR (mL/min/1.73m^2^) | 686 | 21 534 | -0.23 (-0.71,0.25) |

Abbreviation: ADHD: attention-deficit/hyperactivity disorder, HbA1c: haemoglobin A1C, BMI: body mass index, eGFR: estimated glomerular filtration rate, LDLc: low-density lipoprotein cholesterol, HDLc: high-density lipoprotein cholesterol

Interaction term: ADHD*year(s) since first recorded type 2 diabetes diagnosis. n: Number of individuals with one baseline measurement and at least one follow-up measurement for the listed clinical parameters. Model adjusted for year at baseline, age at baseline, sex, other psychiatric disorders history, somatic disorders history

^*^ Statistically significant at a false discovery rate corrected p value <0.0001

# Table S6. Association between ADHD and cardiometabolic risk profile at first recorded type 2 diabetes diagnosis (baseline), where ADHD was defined only by diagnosis at Patient Register using ICD code

| **Clinical parameters** | **n** | | **Mean (SD)** | | **Beta coefficients**  **(95%CI)^*^** |
| --- | --- | --- | --- | --- | --- |
|  | **ADHD** | **Non-ADHD** | **ADHD** | **Non-ADHD** |  |
| HbA1c(%) | 734 | 72 014 | 7.27 (1.82) | 6.98 (1.58) | 0.05 (-0.07,0.17) |
| HbA1c (mmol/mol) |  |  | 55.99 (19.93) | 52.79 (17.30) | 0.51 (-0.80,1.81) |
| Systolic blood pressure (mmHg) | 658 | 67 433 | 132.05 (15.75) | 136.10 (16.68) | 1.18 (-0.14,2.49) |
| Diastolic blood pressure (mmHg) | 659 | 67 344 | 83.24 (10.98) | 82.36 (10.09) | 0.43 (-0.38,1.24) |
| BMI (kg/m^2^) | 582 | 59 171 | 35.52 (7.38) | 31.90 (5.96) | 0.78 (0.30,1.25) |
| Total cholesterol (mmol/L) | 513 | 56 156 | 5.42 (1.42) | 5.33 (1.19) | 0.08 (-0.02,0.19) |
| LDLc (mmol/L) | 458 | 51 301 | 3.37 (1.12) | 3.31 (1.02) | 0.04 (-0.06,0.13) |
| HDLc (mmol/L) | 461 | 51 282 | 1.06 (0.29) | 1.19 (0.36) | -0.02 (-0.05,0.01) |
| Triglycerides (mmol/L) | 616 | 62 705 | 2.78 (2.53) | 2.16 (1.79) | 0.16 (-0.01,0.34) |
| eGFR (mL/min/1.73m^2^) | 438 | 49 589 | 101.11 (26.28) | 91.47 (22.42) | -0.27 (-2.00,1.47) |
| **Behavioural factors** | **n (%)** | | **Risk Ratio (95%CI)** | | |
|  | **ADHD** | **Non-ADHD** |  | | |
| Smoking | N=563 | N=60 707 |  | | |
|  | 206 (37) | 13 042 (21) | 1.15 (1.03,1.29) | | |
| Low physically active | N=426 | N=44 818 |  | | |
|  | 235 (55) | 21 380 (48) | 0.98 (0.89,1.07) | | |

Abbreviation: ADHD: attention-deficit/hyperactivity disorder, HbA1c: haemoglobin A1C, BMI: body mass index, LDLc: low-density lipoprotein cholesterol, HDLc: high-density lipoprotein cholesterol, eGFR: estimated glomerular filtration rate

Target management levels for clinical parameters in individuals with type 2 diabetes : HbA1c: <7% (53 mmol/mol); Diastolic Blood Pressure: <80 mmHg; Systolic Blood Pressure: <130 mmHg; BMI: <25 kg/m²; Total Cholesterol: <4 mmol/L; LDLc: <2.6 mmol/L; HDLc: >1.0 mmol/L; Triglycerides: <1.7 mmol/L; eGFR: ≥90 mL/min/1.73 m²

n: For clinical parameters, refers to number of individuals with corresponding measurements; for behavioural factors, refers to number and proportion of individuals who are smokers or have low physical activity level among those whose smoking status or physical activity levels were measured for ADHD group and non-ADHD group at type 2 diabetes diagnosis. Model adjusted for year at baseline, age at baseline, sex, other psychiatric disorders history, somatic disorders history

^*^ Statistically significant at a false discovery rate corrected p value of <0.0001

# Table S7. Association between ADHD and change in clinical parameters over five years following first recorded type 2 diabetes diagnosis, where ADHD was defined only by diagnosis at Patient Register using ICD code

| **Clinical parameters** | **n** | | **Coefficients for interaction term (95%CI)** |
| --- | --- | --- | --- |
|  | **ADHD** | **Non-ADHD** |  |
| HbA1c (%) | 690 | 67 423 | -0.01 (-0.05,0.04) |
| HbA1c (mmol/mol) |  |  | -0.07 (-0.54,0.40) |
| Systolic blood pressure (mmHg) | 605 | 62 775 | -0.23 (-0.57,0.11) |
| Diastolic blood pressure (mmHg) | 605 | 62 674 | -0.05 (-0.30,0.20) |
| BMI (kg/m^2^) | 539 | 54 932 | -0.25 (-0.34,-0.15)^*^ |
| Total cholesterol (mmol/L) | 458 | 51 197 | 0.01 (-0.03,0.05) |
| LDLc (mmol/L) | 409 | 46 507 | 0.02 (-0.01,0.06) |
| HDLc (mmol/L) | 406 | 46 146 | 0.001 (-0.01,0.01) |
| Triglycerides (mmol/L) | 380 | 43 924 | -0.02 (-0.11,0.07) |
| eGFR (mL/min/1.73m^2^) | 562 | 58 025 | -0.14 (-0.65,0.37) |

Abbreviation: ADHD: attention-deficit/hyperactivity disorder, HbA1c: haemoglobin A1C, BMI: body mass index, eGFR: estimated glomerular filtration rate, LDLc: low-density lipoprotein cholesterol, HDLc: high-density lipoprotein cholesterol

Interaction term: ADHD*year(s) since first recorded type 2 diabetes diagnosis. n: Number of individuals with one baseline measurement and at least one follow-up measurement for the listed clinical parameters. Model adjusted for year at baseline, age at baseline, sex, other psychiatric disorders history, somatic disorders history

^*^ Statistically significant at a false discovery rate corrected p value <0.0001

# Table S8. Association between ADHD and change in behavioural factors two years following first recorded type 2 diabetes diagnosis, where ADHD was defined only by diagnosis at Patient Register using ICD code

| **Behavioural factors** | **n (%)** | | **Risk Ratio (95%CI)^*^** |
| --- | --- | --- | --- |
|  | **ADHD** | **Non-ADHD** |  |
|  | N=99 | N=4 524 |  |
| **Quit smoking** | 13 (13) | 728 (16) | 1.12 (0.64,1.96) |
|  | N=176 | N=18 131 |  |
| **Changed from low to high physically active** | 59 (34) | 7 730 (43) | 0.93 (0.75,1.16) |

Abbreviation: ADHD, attention-deficit/hyperactivity disorder

n: Number and proportion of individuals who had corresponding unhealthy behavioural factor at baseline and had corresponding behavioural factor recorded two years after baseline, changed to healthy behavioural factors two years after baseline among individuals with ADHD or non-ADHD, respectively. For example, there are 99 individuals who were smokers at baseline with ADHD and had smoking status recorded two years after baseline. Among them, 13/99=13% individuals quitted smoking two years after baseline. Model adjusted for calendar year, age and sex at type 2 diabetes diagnosis, other psychiatric disorders history, somatic disorders history

^*^ Statistically significant threshold at a false discovery rate corrected p value <0.0001

# Table S9. Association between ADHD and clinical parameters at first recorded type 2 diabetes diagnosis (baseline), adjusting for baseline BMI

| **Clinical parameters^a^** | **n** | | **Mean (SD)** | | **Beta coefficients**  **(95%CI)^*^** |
| --- | --- | --- | --- | --- | --- |
|  | **ADHD** | **Non-ADHD** | **ADHD** | **Non-ADHD** |  |
| HbA1c (%) | 827 | 56 130 | 7.20 (1.73) | 6.92 (1.51) | 0.05 (-0.05,0.16) |
| HbA1c (mmol/mol) |  |  | 55.17 (18.87) | 52.16 (16.50) | 0.59 (-0.60,1.79) |
| Systolic blood pressure (mmHg) | 768 | 54 081 | 131.85 (15.03) | 135.87 (16.50) | 0.64 (-0.56,1.85) |
| Diastolic blood pressure (mmHg) | 769 | 53 993 | 83.16 (10.48) | 82.21 (10.02) | 0.22 (-0.53,0.96) |
| Total cholesterol (mmol/L) | 610 | 44 631 | 5.37 (1.29) | 5.32 (1.18) | 0.05 (-0.05,0.15) |
| LDLc (mmol/L) | 552 | 40 866 | 3.33 (1.07) | 3.29 (1.01) | 0.01 (-0.08,0.10) |
| HDLc (mmol/L) | 559 | 41 063 | 1.07 (0.33) | 1.20 (0.36) | 0.001 (-0.03,0.03) |
| Triglycerides (mmol/L) | 525 | 39 591 | 2.68 (2.33) | 2.14 (1.76) | 0.10 (-0.05,0.26) |
| eGFR (mL/min/1.73m^2^) | 717 | 48 569 | 100.47 (25.73) | 90.86 (21.95) | 0.20 (-1.40,1.80) |

Abbreviation: ADHD: attention-deficit/hyperactivity disorder, HbA1c: haemoglobin A1C, LDLc: low-density lipoprotein cholesterol, HDLc: high-density lipoprotein cholesterol, eGFR: estimated glomerular filtration rate

Target management levels for clinical parameters in individuals with type 2 diabetes : HbA1c: <7% (53 mmol/mol); Diastolic Blood Pressure: <80 mmHg; Systolic Blood Pressure: <130 mmHg; Total Cholesterol: <4 mmol/L; LDLc: <2.6 mmol/L; HDLc: >1.0 mmol/L; Triglycerides: <1.7 mmol/L; eGFR: ≥90 mL/min/1.73 m²

n: Number of individuals with one baseline measurement and at least one follow-up measurement for the listed clinical parameters. Model adjusted for year at baseline, age at baseline, sex, other psychiatric disorders history, somatic disorders history

^*^ Statistically significant at a false discovery rate corrected p value of <0.0001

# Table S10. Association between ADHD and change in clinical parameters over five years following first recorded type 2 diabetes diagnosis, additionally adjusting for baseline BMI

| **Clinical parameters** | **n** | | **Coefficients for interaction term (95%CI)** |
| --- | --- | --- | --- |
|  | **ADHD** | **Non-ADHD** |  |
| HbA1c (%) | 769 | 52 935 | -0.01 (-0.04,0.03) |
| HbA1c (mmol/mol) |  |  | -0.07 (-0.48,0.35) |
| Systolic blood pressure (mmHg) | 707 | 50 719 | -0.23 (-0.54,0.08) |
| Diastolic blood pressure (mmHg) | 708 | 50 626 | 0.01 (-0.22,0.24) |
| BMI (kg/m^2^) | 799 | 54 672 | -0.26 (-0.34,-0.17)^*^ |
| Total cholesterol (mmol/L) | 544 | 40 888 | 0.03 (-0.01,0.06) |
| LDLc (mmol/L) | 491 | 37 194 | 0.04 (0.01,0.07) |
| HDLc (mmol/L) | 492 | 37 114 | -0.0001 (-0.01,0.01) |
| Triglycerides (mmol/L) | 455 | 35 161 | 0.03 (-0.04,0.10) |
| eGFR (mL/min/1.73m^2^) | 653 | 45 233 | -0.09 (-0.55,0.38) |

Abbreviation: ADHD: attention-deficit/hyperactivity disorder, HbA1c: haemoglobin A1C, BMI: body mass index, eGFR: estimated glomerular filtration rate, LDLc: low-density lipoprotein cholesterol, HDLc: high-density lipoprotein cholesterol

Interaction term: ADHD*year(s) since first recorded type 2 diabetes diagnosis. n: Number of individuals with one baseline measurement and at least one follow-up measurement for the listed clinical parameters. Model adjusted for year at baseline, age at baseline, sex, other psychiatric disorders history, somatic disorders history

^*^ Statistically significant at a false discovery rate corrected p value <0.0001

# Table S11. Association between ADHD and change in clinical parameters over five years following first recorded type 2 diabetes diagnosis, where ADHD examined as a three-category variable

| **Clinical parameters** |  | **n** |  | **Coefficients for interaction term (95%CI)** |
| --- | --- | --- | --- | --- |
|  | **Non-ADHD group (Ref.)** | **ADHD without medication** | **ADHD with medication** |  |
| **HbA1c (%)** |  |  |  |  |
| ADHD without medication vs. Non-ADHD  ADHD with medication vs. Non-ADHD | 67 110 | 730 | 273 | -0.02 (-0.06,0.02)  0.05 (-0.02,0.11) |
| **HbA1c (mmol/mol)** |  |  |  |  |
| ADHD without medication vs. Non-ADHD  ADHD with medication vs. Non-ADHD | 67 110 | 730 | 273 | -0.25 (-0.68,0.18)  0.51 (-0.23,1.25) |
| **Systolic blood pressure (mmHg)** |  |  |  |  |
| ADHD without medication vs. Non-ADHD  ADHD with medication vs. Non-ADHD | 62 489 | 660 | 231 | -0.24 (-0.57,0.08)  -0.18 (-0.81,0.45) |
| **Diastolic blood pressure (mmHg)** |  |  |  |  |
| ADHD without medication vs. Non-ADHD  ADHD with medication vs. Non-ADHD | 62 387 | 661 | 231 | 0.08 (-0.16,0.32)  -0.15 (-0.62,0.31) |
| **BMI (kg/m^2^)** |  |  |  |  |
| ADHD without medication vs. Non-ADHD  ADHD with medication vs. Non-ADHD | 54 672 | 591 | 208 | -0.27 (-0.37,-0.17)^*^  -0.21 (-0.37,-0.06) |
| **Cholesterol (mmol/L)** |  |  |  |  |
| ADHD without medication vs. Non-ADHD  ADHD with medication vs. Non-ADHD | 50 966 | 485 | 204 | 0.03 (-0.01,0.07)  0.02 (-0.03,0.07) |
| **LDLc (mmol/L)** |  |  |  |  |
| ADHD without medication vs. Non-ADHD  ADHD with medication vs. Non-ADHD | 46 302 | 430 | 184 | 0.04 (0.01,0.08)  0.02 (-0.03,0.06) |
| **HDLc (mmol/L)** |  |  |  |  |
| ADHD without medication vs. Non-ADHD  ADHD with medication vs. Non-ADHD | 45 936 | 437 | 179 | -0.002 (-0.01,0.01)  -0.01 (-0.01,0.004) |
| **Triglycerides (mmol/L)** |  |  |  |  |
| ADHD without medication vs. Non-ADHD  ADHD with medication vs. Non-ADHD | 43 728 | 414 | 162 | 0.01 (-0.07,0.10)  -0.01 (-0.09,0.06) |
| **eGFR (mL/min/1.73m^2^)** |  |  |  |  |
| ADHD without medication vs. Non-ADHD  ADHD with medication vs. Non-ADHD | 57 736 | 601 | 250 | -0.11 (-0.57,0.34)  0.24 (-0.69,1.17) |

Abbreviation: ADHD: attention-deficit/hyperactivity disorder, HbA1c: haemoglobin A1C, BMI: body mass index, eGFR: estimated glomerular filtration rate, LDLc: low-density lipoprotein cholesterol, HDLc: high-density lipoprotein cholesterol

Interaction term: ADHD*year(s) since first recorded type 2 diabetes diagnosis. n: Number of individuals with one baseline measurement and at least one follow-up measurement for the listed clinical parameters for individuals without ADHD, with ADHD but didn’t take ADHD medication or with ADHD and took ADHD medication at baseline. Model adjusted for year at baseline, age at baseline, sex, other psychiatric disorders history, somatic disorders history

^*^ Statistically significant at a false discovery rate corrected p value <0.0001

# Table S12. Association between ADHD and change in behavioural factors two years after first recorded type 2 diabetes diagnosis, where ADHD examined as a three-category variable

| **Behavioural factors** |  | **n (%)** |  | **Risk Ratio (95%CI)^*^** |
| --- | --- | --- | --- | --- |
|  | **Non-ADHD (Ref.)** | **ADHD without medication** | **ADHD with medication** |  |
| **Quit smoking** | N=4 482 | N=74 | N=67 |  |
| ADHD without medication vs. Non-ADHD  ADHD with medication vs. Non-ADHD | 724 (16) | 14 (19) | -^a^ | 0.22 (0.07,0.72)  0.62 (0.38,1.02) |
| **Changed from low to high physically active** | N=18 045 | N=129 | N=133 |  |
| ADHD without medication vs. Non-ADHD  ADHD with medication vs. Non-ADHD | 7 700 (43) | 40 (31) | 49 (37) | 1.19 (0.85,1.68)  1.18 (0.91,1.53) |

Abbreviation: ADHD, attention-deficit/hyperactivity disorder

n: Number and proportion of individuals who had corresponding unhealthy behavioural factor at baseline and had corresponding behavioural factor recorded two years after baseline, changed to healthy behavioural factors two years after baseline among individuals without ADHD, with ADHD but didn’t take ADHD medication or with ADHD and took ADHD medication, respectively. For example, there are 4482 individuals who were smokers at baseline without ADHD and had smoking status recorded two years after baseline. Among them , 724/4482=16% individuals quitted smoking two years after baseline. Model adjusted for calendar year, age and sex at type 2 diabetes diagnosis, other psychiatric disorders history, somatic disorders history

^a^ n<10

^*^ Statistically significant threshold at a false discovery rate corrected p value <0.0001

# Table S13. Association between ADHD and cardiometabolic risk profile at type 2 diabetes diagnosis, when excluded study population with history of schizophrenia or bipolar disorder or substance use disorder

|  | **n** | | **Mean (SD)** | | **Beta coefficients (95%CI)^*^** |
| --- | --- | --- | --- | --- | --- |
| **Clinical parameters** | **ADHD** | **non-ADHD** | **ADHD** | **non-ADHD** |  |
| HbA1c(%) | 658 | 66 336 | 7.24 (1.74) | 6.98 (1.57) | -0.01 (-0.13,0.12) |
| HbA1c(mmol/mol) |  |  | 55.59 (18.99) | 52.75 (17.17) | -0.10 (-1.47,1.26) |
| Systolic blood pressure(mmHg) | 607 | 62 144 | 132.27 (15.24) | 136.31 (16.64) | 0.69 (-0.68,2.06) |
| Diastolic blood pressure(mmHg) | 608 | 62 060 | 82.99 (10.73) | 82.37 (10.06) | 0.34 (-0.50,1.18) |
| BMI(kg/m^2^) | 539 | 54 634 | 35.34 (7.28) | 31.84 (5.91) | 0.76 (0.27,1.25) |
| Total cholesterol(mmol/L) | 482 | 51 918 | 5.47 (1.36) | 5.32 (1.18) | 0.17 (0.06,0.28) |
| LDLc (mmol/L) | 438 | 47 450 | 3.38 (1.05) | 3.31 (1.01) | 0.06 (-0.04,0.15) |
| HDLc(mmol/L) | 440 | 47 396 | 1.06 (0.28) | 1.20 (0.36) | -0.02 (-0.05,0.02) |
| Triglycerides(mmol/L) | 415 | 45 829 | 2.74 (2.27) | 2.13 (1.75) | 0.25 (0.08,0.43) |
| eGFR(mL/min/1.73m^2^) | 570 | 57 639 | 101.54 (25.79) | 91.21 (22.20) | 0.55 (-1.23,2.33) |
|  |  | **n (%)** | **Risk Ratio (95%CI)^*^** | | |
| **Behavioural factors** | **ADHD** | **Non-ADHD** |  |  |  |
|  | N=516 | N=56 004 |  | | |
| Smoking | 142 (28) | 11 047 (20) | 1.27 (1.10,1.47) | | |
|  | N=387 | N=41 306 |  | | |
| Low physically active | 207 (53) | 19 525 (47) | 0.96 (0.87,1.06) | | |

Abbreviation: ADHD: attention-deficit/hyperactivity disorder, HbA1c: haemoglobin A1C, BMI: body mass index, LDLc: low-density lipoprotein cholesterol, HDLc: high-density lipoprotein cholesterol, eGFR: estimated glomerular filtration rate

Target management levels for clinical parameters in individuals with type 2 diabetes : HbA1c: <7% (53 mmol/mol); Diastolic Blood Pressure: <80 mmHg; Systolic Blood Pressure: <130 mmHg; BMI: <25 kg/m²; Total Cholesterol: <4 mmol/L; LDLc: <2.6 mmol/L; HDLc: >1.0 mmol/L; Triglycerides: <1.7 mmol/L; eGFR: ≥90 mL/min/1.73 m²

n: For clinical parameters, refers to number of individuals with corresponding measurements; for behavioural factors, refers to number and proportion of individuals who are smokers or have low physical activity level among those whose smoking status or physical activity levels were measured for ADHD group and non-ADHD group at type 2 diabetes diagnosis. Model adjusted for year at baseline, age at baseline, sex, other psychiatric disorders history, somatic disorders history

^*^ Statistically significant at a false discovery rate corrected p value of <0.0001

# Table S14. Association between ADHD and change in clinical parameters over five years following type 2 diabetes diagnosis, when excluded study population with history of schizophrenia or bipolar disorder or substance use disorder

|  | **n** | | **Coefficients for interaction term (95%CI)^*^** |
| --- | --- | --- | --- |
| **Clinical parameters** | **ADHD** | **Non-ADHD** |  |
| HbA1c(%) | 613 | 62 179 | -0.01 (-0.05,0.04) |
| HbA1c(mmol/mol) |  |  | -0.06 (-0.51,0.39) |
| Systolic blood pressure(mmHg) | 565 | 57 941 | -0.22 (-0.56,0.12) |
| Diastolic blood pressure(mmHg) | 565 | 57 844 | 0.06 (-0.19,0.32) |
| BMI(kg/m^2^) | 504 | 50 791 | -0.18 (-0.29,-0.08) |
| Total cholesterol(mmol/L) | 431 | 47 447 | 0.03 (-0.01,0.07) |
| LDLc (mmol/L) | 392 | 43 103 | 0.04 (0.002,0.07) |
| HDLc(mmol/L) | 390 | 42 738 | 0.002 (-0.01,0.01) |
| Triglycerides(mmol/L) | 368 | 40 694 | -0.01 (-0.06,0.05) |
| eGFR(mL/min/1.73m^2^) | 520 | 53 452 | 0.10 (-0.39,0.59) |

Abbreviation: ADHD: attention-deficit/hyperactivity disorder, HbA1c: haemoglobin A1C, BMI: body mass index, eGFR: estimated glomerular filtration rate, LDLc: low-density lipoprotein cholesterol, HDLc: high-density lipoprotein cholesterol

Interaction term: ADHD*year(s) since first recorded type 2 diabetes diagnosis. n: Number of individuals with one baseline measurement and at least one follow-up measurement for the listed clinical parameters. Model adjusted for year at baseline, age at baseline, sex, other psychiatric disorders history, somatic disorders history

^*^ Statistically significant at a false discovery rate corrected p value <0.0001

# Table S15. Association between ADHD and change in behavioural factors two years after type 2 diabetes diagnosis, when excluded study population with history of schizophrenia or bipolar disorder or substance use disorder

|  | **n (%)** | | **Risk Ratio (95%CI)^*^** |
| --- | --- | --- | --- |
| **Behavioural factors change** | **ADHD** | **Non-ADHD** |  |
|  | N=66 | N=3 796 |  |
| Quit smoking | 15 (23) | 660 (17) | -^a^ |
|  | N=161 | N=16 708 |  |
| Changed from low to high physically active | 56 (35) | 7 207 (43) | 0.87 (0.69,1.08) |

Abbreviation: ADHD, attention-deficit/hyperactivity disorder

n: Number and proportion of individuals who had corresponding unhealthy behavioural factor at baseline and had corresponding behavioural factor recorded two years after baseline, changed to healthy behavioural factors two years after baseline among individuals with ADHD or non-ADHD, respectively. For example, there are 66 individuals who were smokers at baseline with ADHD and had smoking status recorded two years after baseline. Among them, 15/66=23% individuals quitted smoking two years after baseline. Model adjusted for calendar year, age and sex at type 2 diabetes diagnosis, other psychiatric disorders history, somatic disorders history

^a^ n<10

^*^ Statistically significant threshold at a false discovery rate corrected p value <0.0001

# Table S16. Association between ADHD and cardiometabolic risk profile at type 2 diabetes diagnosis, when further adjusting for psychotropic medication use within 3 months before type 2 diabetes diagnosis

|  | **n** | | **Mean (SD)** | | **Beta coefficients (95%CI)** | |
| --- | --- | --- | --- | --- | --- | --- |
| **Clinical parameters** | **ADHD** | **non-ADHD** | **ADHD** | **non-ADHD** | **Model 4** | **Model 5** |
| HbA1c(%) | 1049 | 63 821 | 7.23 (1.78) | 6.99 (1.61) | 0.003 (-0.10,0.11) | -0.004 (-0.11,0.10) |
| HbA1c(mmol/mol) |  |  | 55.49 (19.41) | 52.87 (17.56) | 0.03 (-1.09,1.16) | -0.05 (-1.17,1.08) |
| Systolic blood pressure(mmHg) | 938 | 59 573 | 132.41 (15.36) | 135.79 (16.53) | 1.34 (0.23,2.45) | 1.23 (0.12,2.35) |
| Diastolic blood pressure(mmHg) | 939 | 59 496 | 83.42 (10.49) | 82.46 (10.15) | 0.54 (-0.15,1.23) | 0.55 (-0.15,1.24) |
| BMI(kg/m^2^) | 838 | 52 060 | 35.48 (7.23) | 32.02 (5.99) | 0.86 (0.45,1.26)^*^ | 0.89 (0.49,1.30)^*^ |
| Total cholesterol(mmol/L) | 759 | 50 830 | 5.41 (1.36) | 5.33 (1.19) | 0.05 (-0.04,0.14) | 0.07 (-0.02,0.16) |
| LDLc (mmol/L) | 679 | 46 703 | 3.36 (1.09) | 3.32 (1.02) | 0.02 (-0.06,0.10) | 0.03 (-0.05,0.11) |
| HDLc(mmol/L) | 687 | 46 484 | 1.08 (0.33) | 1.19 (0.36) | -0.01 (-0.03,0.02) | -0.01 (-0.03,0.02) |
| Triglycerides(mmol/L) | 647 | 44 660 | 2.72 (2.36) | 2.15 (1.79) | 0.12 (-0.03,0.26) | 0.14 (-0.01,0.28) |
| eGFR(mL/min/1.73m^2^) | 911 | 57 075 | 100.26 (25.30) | 92.24 (22.45) | -0.59 (-2.05,0.86) | -0.67 (-2.12,0.79) |
|  | **n (%)** | | **Risk Ratio (95%CI)** | | | |
| **Behavioural factors** | **ADHD** | **Non-ADHD** | **Model 4** | | **Model 5** | |
|  | N=811 | N=52 993 |  | |  | |
| Smoking | 290 (36) | 11 429 (22) | 1.10 (0.99,1.22) | | 1.12 (1.01,1.24) | |
|  | N=628 | N=43 352 |  | |  | |
| Low physically active | 345 (55) | 20 659 (48) | 0.98 (0.90,1.05) | | 0.99 (0.91,1.07) | |

Abbreviation: ADHD: attention-deficit/hyperactivity disorder, HbA1c: haemoglobin A1C, BMI: body mass index, LDLc: low-density lipoprotein cholesterol, HDLc: high-density lipoprotein cholesterol, eGFR: estimated glomerular filtration rate

Target management levels for clinical parameters in individuals with type 2 diabetes : HbA1c: <7% (53 mmol/mol); Diastolic Blood Pressure: <80 mmHg; Systolic Blood Pressure: <130 mmHg; BMI: <25 kg/m²; Total Cholesterol: <4 mmol/L; LDLc: <2.6 mmol/L; HDLc: >1.0 mmol/L; Triglycerides: <1.7 mmol/L; eGFR: ≥90 mL/min/1.73 m²

n: For clinical parameters, refers to number of individuals with corresponding measurements; for behavioural factors, refers to number and proportion of individuals who are smokers or have low physical activity level among those whose smoking status or physical activity levels were measured for ADHD group and non-ADHD group at type 2 diabetes diagnosis. Model 4: adjusted for calendar year, age and sex at type 2 diabetes diagnosis, other psychiatric disorders history, somatic disorders history, and use of antipsychotics, antidepressants and mood stabilizer within 3 months before type 2 diabetes diagnosis; Model 5: adjusted for calendar year, age and sex at type 2 diabetes diagnosis, other psychiatric disorders history, somatic disorders history, and use of second-generation antipsychotics (N05AD03, N05AE03, N05AE04, N05AE05, N05AH02, N05AH03, N05AH04, N05AL01, N05AL05, N05AX08, N05AX12, N05AX13, N05AX15) within 3 months before type 2 diabetes diagnosis

^*^ Statistically significant at a false discovery rate corrected p value of <0.0001

# Table S17. Association between ADHD and change in clinical parameters over five years following type 2 diabetes diagnosis, when further adjusting for psychotropic medication use within 3 months before type 2 diabetes diagnosis

|  | **n** | | **Coefficients for interaction term (95%CI)** | |
| --- | --- | --- | --- | --- |
| **Clinical parameters** | **ADHD** | **Non-ADHD** | **Model 4** | **Model 5** |
| HbA1c(%) | 975 | 60 328 | -0.003 (-0.04,0.03) | -0.003 (-0.04,0.03) |
| HbA1c(mmol/mol) |  |  | -0.03 (-0.41,0.35) | -0.03 (-0.41,0.35) |
| Systolic blood pressure(mmHg) | 861 | 55 993 | -0.24 (-0.53,0.06) | -0.24 (-0.53,0.06) |
| Diastolic blood pressure(mmHg) | 862 | 55 913 | 0.02 (-0.19,0.24) | 0.02 (-0.19,0.24) |
| BMI(kg/m^2^) | 774 | 48 746 | -0.25 (-0.34,-0.16)^d^ | -0.25 (-0.34,-0.16)^*^ |
| Total cholesterol(mmol/L) | 669 | 46 526 | 0.03 (-0.01,0.06) | 0.03 (-0.01,0.06) |
| LDLc (mmol/L) | 598 | 42 589 | 0.04 (0.01,0.06) | 0.04 (0.01,0.06) |
| HDLc(mmol/L) | 598 | 42 028 | -0.003 (-0.01,0.004) | -0.003 (-0.01,0.004) |
| Triglycerides(mmol/L) | 558 | 39 696 | 0.01 (-0.06,0.07) | 0.01 (-0.06,0.07) |
| eGFR(mL/min/1.73m^2^) | 828 | 53 048 | 0.05 (-0.38,0.47) | 0.05 (-0.37,0.47) |

Abbreviation: ADHD: attention-deficit/hyperactivity disorder, HbA1c: haemoglobin A1C, BMI: body mass index, eGFR: estimated glomerular filtration rate, LDLc: low-density lipoprotein cholesterol, HDLc: high-density lipoprotein cholesterol

Interaction term: ADHD*year(s) since first recorded type 2 diabetes diagnosis. n: Number of individuals with one baseline measurement and at least one follow-up measurement for the listed clinical parameters. Model 4: adjusted for calendar year, age and sex at type 2 diabetes diagnosis, other psychiatric disorders history, somatic disorders history, and use of antipsychotics, antidepressants and mood stabilizer within 3 months before type 2 diabetes diagnosis; Model 5: adjusted for calendar year, age and sex at type 2 diabetes diagnosis, other psychiatric disorders history, somatic disorders history, and use of second-generation antipsychotics (N05AD03, N05AE03, N05AE04, N05AE05, N05AH02, N05AH03, N05AH04, N05AL01, N05AL05, N05AX08, N05AX12, N05AX13, N05AX15) within 3 months before type 2 diabetes diagnosis

^*^ Statistically significant at a false discovery rate corrected p value of <0.0001

# Table S18. Association between ADHD and change in behavioural factors two years after type 2 diabetes diagnosis, when further adjusting for psychotropic medication use within 3 months before type 2 diabetes diagnosis

|  | **n (%)** | | **Risk Ratio (95%CI)^*^** | |
| --- | --- | --- | --- | --- |
| **Behavioural factors change** | **ADHD** | **Non-ADHD** | **Model 4** | **Model 5** |
|  | N=141 | N=4 481 |  |  |
| Quit smoking | 17 (12) | 724 (16) | 0.97 (0.59,1.60) | 0.99 (0.61,1.61) |
|  | N=256 | N=17 480 |  |  |
| Changed from low to high physically active | 87 (34) | 7 476 (43) | 0.93 (0.78,1.12) | 0.93 (0.77,1.11) |

Abbreviation: ADHD, attention-deficit/hyperactivity disorder

n: Number and proportion of individuals who had corresponding unhealthy behavioural factor at baseline and had corresponding behavioural factor recorded two years after baseline, changed to healthy behavioural factors two years after baseline among individuals with ADHD or non-ADHD, respectively. For example, there are 141 individuals who were smokers at baseline with ADHD and had smoking status recorded two years after baseline. Among them, 17/141=12% individuals quitted smoking two years after baseline. Model 4: adjusted for calendar year, age and sex at type 2 diabetes diagnosis, other psychiatric disorders history, somatic disorders history, and use of antipsychotics, antidepressants and mood stabilizer within 3 months before type 2 diabetes diagnosis; Model 5: adjusted for calendar year, age and sex at type 2 diabetes diagnosis, other psychiatric disorders history, somatic disorders history, and use of second-generation antipsychotics (N05AD03, N05AE03, N05AE04, N05AE05, N05AH02, N05AH03, N05AH04, N05AL01, N05AL05, N05AX08, N05AX12, N05AX13, N05AX15) within 3 months before type 2 diabetes diagnosis

^*^ Statistically significant at a false discovery rate corrected p value of <0.0001

# Table S19. Association between ADHD and cardiometabolic risk profile at type 2 diabetes diagnosis, when examining ADHD status within 5 years before type 2 diabetes diagnosis

|  | **n** | | **Mean (SD)** | | **Beta coefficients (95%CI)^*^** |
| --- | --- | --- | --- | --- | --- |
| **Clinical parameters** | **ADHD** | **non-ADHD** | **ADHD** | **non-ADHD** |  |
| HbA1c(%) | 290 | 72 458 | 7.10 (1.67) | 6.98 (1.59) | -0.10 (-0.29,0.08) |
| HbA1c(mmol/mol) |  |  | 54.14 (18.30) | 52.81 (17.33) | -1.12 (-3.12,0.88) |
| Systolic blood pressure(mmHg) | 266 | 67 825 | 130.44 (15.11) | 136.08 (16.68) | -0.79 (-2.79,1.22) |
| Diastolic blood pressure(mmHg) | 266 | 67 737 | 82.78 (10.82) | 82.36 (10.10) | -0.10 (-1.33,1.13) |
| BMI(kg/m^2^) | 226 | 59 527 | 34.77 (7.05) | 31.92 (5.98) | 0.09 (-0.64,0.82) |
| Total cholesterol(mmol/L) | 220 | 56 449 | 5.47 (1.46) | 5.33 (1.19) | 0.14 (-0.01,0.30) |
| LDLc (mmol/L) | 190 | 51 569 | 3.42 (1.13) | 3.31 (1.02) | 0.12 (-0.02,0.26) |
| HDLc(mmol/L) | 196 | 51 547 | 1.09 (0.29) | 1.19 (0.36) | 0.01 (-0.04,0.06) |
| Triglycerides(mmol/L) | 187 | 49 840 | 2.76 (2.50) | 2.16 (1.79) | 0.14 (-0.12,0.40) |
| eGFR(mL/min/1.73m^2^) | 247 | 63 074 | 99.99 (26.46) | 91.53 (22.46) | -0.05 (-2.70,2.59) |
|  | **n (%)** | | **Risk Ratio (95%CI)^*^** | | |
| **Behavioural factors** | **ADHD** | **Non-ADHD** |  |  |  |
|  | N=214 | N=61 056 |  | | |
| Smoking | 81 (38) | 13 167 (22) | 1.18 (1.00,1.39) | | |
|  | N=164 | N=45 080 |  | | |
| Low physically active | 99 (60) | 21 516 (48) | 1.08 (0.95,1.23) | | |

Abbreviation: ADHD: attention-deficit/hyperactivity disorder, HbA1c: haemoglobin A1C, BMI: body mass index, LDLc: low-density lipoprotein cholesterol, HDLc: high-density lipoprotein cholesterol, eGFR: estimated glomerular filtration rate

Target management levels for clinical parameters in individuals with type 2 diabetes : HbA1c: <7% (53 mmol/mol); Diastolic Blood Pressure: <80 mmHg; Systolic Blood Pressure: <130 mmHg; BMI: <25 kg/m²; Total Cholesterol: <4 mmol/L; LDLc: <2.6 mmol/L; HDLc: >1.0 mmol/L; Triglycerides: <1.7 mmol/L; eGFR: ≥90 mL/min/1.73 m²

n: For clinical parameters, refers to number of individuals with corresponding measurements; for behavioural factors, refers to number and proportion of individuals who are smokers or have low physical activity level among those whose smoking status or physical activity levels were measured for ADHD group and non-ADHD group at type 2 diabetes diagnosis. Model adjusted for year at baseline, age at baseline, sex, other psychiatric disorders history, somatic disorders history

^*^ Statistically significant at a false discovery rate corrected p value of <0.0001

# Table S20. Association between ADHD and change in clinical parameters over five years following type 2 diabetes diagnosis, when examining ADHD status within 5 years before type 2 diabetes diagnosis

|  | **n** | | **Coefficients for interaction term (95%CI)^*^** |
| --- | --- | --- | --- |
| **Clinical parameters** | **ADHD** | **Non-ADHD** |  |
| HbA1c(%) | 271 | 67 842 | 0.05 (-0.02,0.13) |
| HbA1c(mmol/mol) |  |  | 0.56 (-0.25,1.37) |
| Systolic blood pressure(mmHg) | 243 | 63 137 | 0.28 (-0.22,0.79) |
| Diastolic blood pressure(mmHg) | 243 | 63 036 | 0.07 (-0.29,0.43) |
| BMI(kg/m^2^) | 204 | 55 267 | -0.19 (-0.32,-0.05) |
| Total cholesterol(mmol/L) | 191 | 51 464 | 0.04 (-0.02,0.11) |
| LDLc (mmol/L) | 166 | 46 750 | 0.05 (0.0001,0.10) |
| HDLc(mmol/L) | 171 | 46 381 | -0.01 (-0.02,0.01) |
| Triglycerides(mmol/L) | 163 | 44 141 | 0.03 (-0.13,0.19) |
| eGFR(mL/min/1.73m^2^) | 229 | 58 358 | -0.07 (-0.93,0.79) |

Abbreviation: ADHD: attention-deficit/hyperactivity disorder, HbA1c: haemoglobin A1C, BMI: body mass index, eGFR: estimated glomerular filtration rate, LDLc: low-density lipoprotein cholesterol, HDLc: high-density lipoprotein cholesterol

Interaction term: ADHD*year(s) since first recorded type 2 diabetes diagnosis. n: Number of individuals with one baseline measurement and at least one follow-up measurement for the listed clinical parameters. Model adjusted for year at baseline, age at baseline, sex, other psychiatric disorders history, somatic disorders history

^*^ Statistically significant at a false discovery rate corrected p value <0.0001

# Table S21. Association between ADHD and change in behavioural factors two years after type 2 diabetes diagnosis, when examining ADHD status within 5 years before type 2 diabetes diagnosis

|  | **n (%)** | | **Risk Ratio (95%CI)^*^** |
| --- | --- | --- | --- |
| **Behavioural factors change** | **ADHD** | **Non-ADHD** |  |
|  | N=40 | N=4 583 |  |
| Quit smoking | -^a^ (-) | 737 (16) | 0.79 (0.31,2.07) |
|  | N=72 | N=18 235 |  |
| Changed from low to high physically active | 23 (32) | 7 766 (43) | 0.90 (0.64,1.26) |

Abbreviation: ADHD, attention-deficit/hyperactivity disorder

n: Number and proportion of individuals who had corresponding unhealthy behavioural factor at baseline and had corresponding behavioural factor recorded two years after baseline, changed to healthy behavioural factors two years after baseline among individuals with ADHD or non-ADHD, respectively. For example, there are 72 individuals who were physically active at baseline with ADHD and had physical activity level recorded two years after baseline. Among them, 23/72=32% individuals became physically active two years after baseline.Model adjusted for calendar year, age and sex at type 2 diabetes diagnosis, other psychiatric disorders history, somatic disorders history

^a^ n <10

^*^ Statistically significant at a false discovery rate corrected p value <0.0001

# Table S22. Comparison of standardized mean differences before and after applying inverse probability of treatment weighting

| **Variables** | **SMDs before IPTW^b^** | **SMDs after IPTW^b^** |
| --- | --- | --- |
| **Age at first recorded T2D (years)** | 1.02 | 0.23 |
| **Year at first recorded T2D (years)** | 0.47 | 0.12 |
| **Female** | **0.03** | **0.02** |
| **Somatic comorbidities** |  |  |
| Obesity^a^ | 0.34 | **0.04** |
| Cardiovascular diseases | **0.07** | **0.03** |
| Hyperlipidaemia | 0.23 | **0.02** |
| Sleep Disorder | 0.31 | 0.18 |
| **Psychiatric comorbidities** |  |  |
| Anxiety disorder | 0.57 | 0.13 |
| Autism spectrum disorder | 0.58 | **0.03** |
| Bipolar disorder | 0.49 | 0.16 |
| Conduct disorder | 0.16 | **0.02** |
| Major depressive disorder | 0.95 | 0.21 |
| Eating disorders | 0.16 | **0.04** |
| Intellectual disability | 0.31 | **0.05** |
| Personality disorders | 0.56 | 0.16 |
| Schizophrenia and psychotic disorders | 0.25 | 0.16 |
| Substance use disorders | 0.71 | 0.16 |

Abbreviations: ADHD: attention-deficit/hyperactivity disorder; T2D: type 2 diabetes; IQR: interquartile range; SMD: standardized mean differences; IPTW: inverse probability of treatment weighting

^a^ Clinically diagnosed obesity requiring specialised care

^b^ An SMD <0.1 was indicative of adequate balance between ADHD and non-ADHD group

# Table S23. Association between ADHD and cardiometabolic risk profile at first recorded type 2 diabetes diagnosis (baseline), after applying inverse probability of treatment weighting

| **Clinical parameters** | **Beta coefficients (95%CI)** |
| --- | --- |
| HbA1c(%) | -0.01(-0.17,0.16) |
| HbA1c(mmol/mol) | -0.08(-1.86,1.71) |
| Systolic blood pressure(mmHg) | -2.59(-4.47,-0.71) |
| Diastolic blood pressure(mmHg) | 0.35(-0.73,1.43) |
| BMI(kg/m^2^) | 1.04(0.22,1.86) |
| Total cholesterol(mmol/L) | 0.12(-0.05,0.29) |
| LDLc (mmol/L) | 0.08(-0.07,0.23) |
| HDLc(mmol/L) | -0.01(-0.06,0.03) |
| Triglycerides(mmol/L) | 0.14(-0.10,0.38) |
| eGFR(mL/min/1.73m^2^) | 1.08(-1.35,3.50) |
| **Behavioural factors** | **Risk Ratio (95%CI)** |
| Smoking | 1.52(1.27,1.83)^*^ |
| Low physically active | 0.99(0.84,1.15) |

Abbreviation: ADHD: attention-deficit/hyperactivity disorder, HbA1c: haemoglobin A1C, BMI: body mass index, LDLc: low-density lipoprotein cholesterol, HDLc: high-density lipoprotein cholesterol, eGFR: estimated glomerular filtration rate

^*^ Statistically significant at a false discovery rate corrected p value of <0.0001

# Table S24. Association between ADHD and change in clinical parameters over five years following first recorded type 2 diabetes diagnosis, after applying inverse probability of treatment weighting

| **Clinical parameters** | **Coefficients for interaction term (95%CI)** |
| --- | --- |
| HbA1c(%) | 0.01(-0.04,0.06) |
| HbA1c(mmol/mol) | 0.13(-0.42,0.68) |
| Systolic blood pressure(mmHg) | -0.30(-1.19,0.58) |
| Diastolic blood pressure(mmHg) | -0.26(-0.94,0.41) |
| BMI(kg/m^2^) | -0.05(-0.17,0.07) |
| Total cholesterol(mmol/L) | 0.02(-0.03,0.06) |
| LDLc (mmol/L) | -0.00(-0.04,0.04) |
| HDLc(mmol/L) | -0.00(-0.01,0.01) |
| Triglycerides(mmol/L) | -0.01(-0.08,0.06) |
| eGFR(mL/min/1.73m^2^) | 0.74(-0.05,1.54) |

Abbreviation: ADHD: attention-deficit/hyperactivity disorder, HbA1c: haemoglobin A1C, BMI: body mass index, eGFR: estimated glomerular filtration rate, LDLc: low-density lipoprotein cholesterol, HDLc: high-density lipoprotein cholesterol

Interaction term: ADHD*year(s) since first recorded type 2 diabetes diagnosis.

^*^ Statistically significant at a false discovery rate corrected p value <0.0001

# Table S25. Association between ADHD and change in behavioural factors two years following first recorded type 2 diabetes diagnosis, after applying inverse probability of treatment weighting

| **Behavioural factors change** | **Risk Ratio (95%CI)*** |
| --- | --- |
| Quit smoking | 0.98(0.50,1.92) |
| Changed from low to high physically active | 0.80(0.58,1.10) |

Abbreviation: ADHD, attention-deficit/hyperactivity disorder

^*^ Statistically significant threshold at a false discovery rate corrected p value <0.0001

# Table S26. Characteristics of individuals with and without ADHD in the cohort of individuals with type 2 diabetes at first recorded type 2 diabetes diagnosis (baseline), stratified by birth cohort and sex

|  | **1930-1959** | | **1960-2002** | | **Male** | | **Female** | |
| --- | --- | --- | --- | --- | --- | --- | --- | --- |
|  | **ADHD (N=225)** | **Non-ADHD (N=49 929)** | **ADHD (N=979)** | **Non-ADHD (N=29 474)** | **ADHD (N=723)** | **Non-ADHD  (N=46 515)** | **ADHD (N=481)** | **Non-ADHD (N=32 888)** |
| **Age at first recorded type 2 diabetes diagnosis (years)**, median (IQR) | 59.0  (55.7,62.3) | 60.6  (57.1,63.4) | 42.7  (33.8,49.2) | 47.8  (42.0,52.4) | 46.1  (37.2,53.8) | 57.0  (50.1,61.8) | 44.9  (34.3,52.2) | 56.9  (49.6,61.8) |
| **Somatic disorders history**, n (%) |  |  |  |  |  |  |  |  |
| Obesity | 21 (9.3) | 2 302 (4.6) | 187 (19.1) | 2 783 (9.4) | 95 (13.1) | 2 121 (4.6) | 113 (23.5) | 2 964 (9.0) |
| Cardiovascular diseases | 98 (43.6) | 17 038 (34.1) | 209 (21.3) | 5 731 (19.4) | 196 (27.1) | 14 436 (31.0) | 111 (23.1) | 8 333 (25.3) |
| Hyperlipidaemia | 77 (34.2) | 16 935 (33.9) | 151 (15.4) | 5 674 (19.3) | 147 (20.3) | 14 223 (30.6) | 81 (16.8) | 8 386 (25.5) |
| Sleep Disorder | 46 (20.4) | 3 505 (7.0) | 159 (16.2) | 2 134 (7.2) | 138 (19.1) | 4 219 (9.1) | 67 (13.9) | 1 420 (4.3) |
| **Psychiatric disorders history**, n (%) |  |  |  |  |  |  |  |  |
| Anxiety disorder | 44 (19.6) | 925 (1.9) | 195 (19.9) | 1 025 (3.5) | 123 (17.0) | 859 (1.8) | 116 (24.1) | 1 091 (3.3) |
| Autism spectrum disorder | 17 (7.6) | 34 (0.1) | 163 (16.6) | 174 (0.6) | 110 (15.2) | 137 (0.3) | 70 (14.6) | 71 (0.2) |
| Bipolar disorder | 29 (12.9) | 486 (1.0) | 134 (13.7) | 440 (1.5) | 81 (11.2) | 397 (0.9) | 82 (17.0) | 529 (1.6) |
| Conduct disorder | 1 (0.4) | 7 (0.01) | 16 (1.6) | 24 (0.1) | 10 (1.4) | 15 (0.03) | 7 (1.5) | 16 (0.04) |
| Major Depressive disorder | 103 (45.8) | 3 027 (6.1) | 435 (44.4) | 2 540 (8.6) | 299 (41.4) | 2 544 (5.5) | 239 (49.7) | 3 023 (9.2) |
| Eating disorders | 1 (0.4) | 20 (0.04) | 18 (1.8) | 75 (0.3) | 3 (0.4) | 12 (0.02) | 16 (3.3) | 83 (0.3) |
| Intellectual disability | 3 (1.3) | 174 (0.3) | 70 (7.2) | 323 (1.1) | 42 (5.8) | 238 (0.5) | 31 (6.4) | 259 (0.8) |
| Personality disorders | 32 (14.2) | 698 (1.4) | 179 (18.3) | 685 (2.3) | 108 (14.9) | 681 (1.5) | 103 (21.4) | 702 (2.1) |
| Schizophrenia and psychotic disorders | 17 (7.6) | 901 (1.8) | 79 (8.1) | 1 010 (3.4) | 61 (8.4) | 1 025 (2.2) | 35 (7.3) | 886 (2.7) |
| Substance use disorders | 69 (30.7) | 2 479 (5.0) | 300 (30.6) | 1 549 (5.3) | 241 (33.3) | 2 806 (6.0) | 128 (26.6) | 1 222 (3.7) |

Abbreviation: ADHD: attention-deficit/hyperactivity disorder

**References**

1 Ludvigsson JF, Otterblad-Olausson P, Pettersson BU, Ekbom A. The Swedish personal identity number: Possibilities and pitfalls in healthcare and medical research. *Eur J Epidemiol* 2009; **24**: 659–67. doi: 10.1007/s10654-009-9350-y.

2 Ludvigsson JF, Almqvist C, Bonamy AKE, Ljung R, Michaëlsson K, Neovius M, *et al.* Registers of the Swedish total population and their use in medical research. *Eur J Epidemiol* 2016; **31**: 125–36. doi: 10.1007/s10654-016-0117-y.

3 Ludvigsson JF, Andersson E, Ekbom A, Feychting M, Kim JL, Reuterwall C, *et al.* External review and validation of the Swedish national inpatient register. *BMC Public Health* 2011; **11**: 450. doi: 10.1186/1471-2458-11-450.

4 Wettermark B, Hammar N, Fored CM, Leimanis A, Olausson PO, Bergman U, *et al.* The new Swedish Prescribed Drug Register--opportunities for pharmacoepidemiological research and experience from the first six months. *Pharmacoepidemiol Drug Saf* 2007; **16**: 726–35. doi: 10.1002/pds.1294.

5 Svensson AM, Eliasson B, Linder E, Almskog I, Hermansson-Carter V, Eeg-Olofsson K, *et al.* *Nationwide results 1996-2020*. Swedish National Diabetes Register, 2021. doi: 10.18158/ryNUNVPiu.
