# Supplementary material for: ADHD and cardiometabolic risk profile in adults with type 2 diabetes: a longitudinal register-based study
Source: BMJ Open. 2026 Jul 3;16(7):e113372. doi: 10.1136/bmjopen-2025-113372 (PMC13343064; doi:10.1136/bmjopen-2025-113372)
Supplement: Supplementary file 2 [file bmjopen-2025-113372.reviewer_comments.pdf]

PEER REVIEW HISTORY

BMJ Open publishes all reviews undertaken for accepted manuscripts. Reviewers are asked to complete a checklist review form and are provided with free text boxes to elaborate on their assessment. These free text comments are reproduced below.

ARTICLE DETAILS

Title (Provisional)

ADHD and Cardiometabolic Risk Profile in Adults with Type 2 Diabetes: A Longitudinal Register-Based Study

Authors

Dong, Zihan; Liu, Shengxin; Lundholm, Cecilia; Brikell, Isabell; Kuja-Halkola, Ralf; D'Onofrio, Brian M; Lichtenstein, Paul; Butwicka, Agnieszka; Gudbjörnsdottir, Soffia; Larsson, Henrik; Chang, Zheng; Du Rietz, Ebba

---

VERSION 1 - REVIEW

**Reviewer**	**1**
**Name**	**Gong, Qiyong**
**Affiliation**	**West China Xiamen Hospital of Sichuan University**
**Date**	**04-Jan-2026**
**COI**	**None**

This manuscript investigated the impact of comorbid ADHD on cardiometabolic risk profiles and their longitudinal changes in adults with type 2 diabetes based on Swedish national registry data. The study has the advantages of a large sample size, well-structured follow-up and reliable data sources. However, several aspects of the study design and interpretation of results require clarification:

1. As shown in Table 1, there are significant differences between the ADHD and non-ADHD groups across key covariates, such as median age (45.6 years vs. 56.9 years), obesity rate (17.3% vs. 6.4%), and the prevalence of various psychiatric comorbidities. The manuscript only provides descriptive statistics and lacks a systematic assessment of the comparability between the groups.

2. The sample size ratio between the exposed group (ADHD, n = 1,204) and the non-exposed group (n = 79,403) is approximately 1:66. With such significant imbalances and clear baseline differences, relying on traditional multivariate regression for covariate adjustment may be difficult to adequately eliminate residual confounding, especially for cardiometabolic measures closely related to age and obesity rates.

3. Table 2 showed that the ADHD group had lower systolic blood pressure and higher eGFR. However, the ADHD group was on average over 11 years younger, and age is a strong determinant of both blood pressure and renal function. Although was age is adjusted in the model, the current analysis may not completely peel off the impact of age differences on these results, and it is recommended to interpret the results more cautiously.

4. Table 1 shows that the obesity rate at T2D diagnosis was significantly higher in the ADHD group compared to the non-ADHD group (17.3% vs. 6.4%). Individuals with a higher baseline BMI or obesity rate often have greater potential for reduction. Although baseline BMI was adjusted for in a sensitivity analysis (Table S10), such a marked baseline disparity suggests the two groups originated from different metabolic risk structures and clinical management contexts. The authors need to be more cautious when interpreting longitudinal BMI changes.

5. The study required that all metabolic measurements be completed within one month after T2D diagnosis (lines 113-114). This requirement poses a higher threshold for individuals with ADHD, who may experience challenges with executive function and organization. This could lead to the selected ADHD cohort being a subgroup with better adherence and potentially less severe conditions, affecting the sample's representativeness and the validity of baseline comparisons. This potential bias has not been discussed in the manuscript.

**Reviewer**	**2**
**Name**	**Okada, Seigo**
**Affiliation**	**Yamaguchi University School of Medicine Graduate School of Medicine**
**Date**	**10-Feb-2026**
**COI**	**None**

This manuscript examines the association between attention-deficit/hyperactivity disorder (ADHD) and cardiometabolic risk profiles among adults with newly diagnosed type 2 diabetes (T2D) using Swedish nationwide register data. The study benefits from a large sample size, high-quality registry data, and extensive sensitivity analyses. The authors have also appropriately acknowledged several methodological strengths and limitations.

However, despite these merits, I have substantial concerns regarding the interpretation of the findings and the validity of the conclusions. In particular, the main conclusions appear to overstate the clinical and conceptual implications of relatively modest and inconsistent associations. While the authors correctly describe the limitations of their data, these limitations are not adequately reflected in the final interpretation. Overall, I am not convinced that the current results sufficiently support the authors’ key claims or justify the proposed clinical implications.

While the dataset is large and well curated, the conceptual advance of the study appears limited. The findings largely confirm previously reported associations between ADHD, obesity, and smoking in the general population and do not substantially extend current knowledge regarding T2D management. Given the absence of strong or consistent effects across most cardiometabolic domains, it is unclear whether this study meaningfully changes existing clinical or scientific understanding.

**Reviewer**	**3**
**Name**	**Nakata, Mitsuko**
**Affiliation**	**Kyoto Prefectural University of Medicine, Department of Biostatistics**
**Date**	**15-Mar-2026**
**COI**	**None**

This is a well-structured, large-scale study that clearly delineates the impact of ADHD on biomarkers and lifestyles at the time of diagnosis as well as various clinical indicators following the initiation of treatment. I have several concerns from a statistical perspective that I would like the authors to address. This review focuses on the statistical methods and analyses described in the manuscript. I have not reviewed the scientific content related to the topic itself, as it falls outside my field of expertise.

【major】

1. Why is the significance level set so low at 0.0001(One-five-hundredth of the conventional significance level)? Please explain.

2. Please provide a clearer description of the GEE model specifications in the Statistical Analysis section, including the choice of the correlation structure and the inclusion of the interaction term between ADHD and time. Especially, please specify how time was modeled: was the linear regression fitted against the number of post-diagnosis measurements or the actual number of years elapsed?

3. Were non-linear models considered for the relationship between time and BMI changes? Please provide more details in the Statistical Analysis section.

【minor】

□ Line 238: Based on Table S5, the beta coefficients for females appear to be smaller than those for males. Could you please verify this?

□ Line 334: Please also discuss the imbalance in birth cohorts observed in Table 1.

VERSION 1 - AUTHOR RESPONSE

Reviewer: 1

Dr. Qiyong Gong, West China Xiamen Hospital of Sichuan University

Comments to the Author:
This manuscript investigated the impact of comorbid ADHD on cardiometabolic risk profiles and their longitudinal changes in adults with type 2 diabetes based on Swedish national registry data. The study has the advantages of a large sample size, well-structured follow-up and reliable data sources. However, several aspects of the study design and interpretation of results require clarification:

1. As shown in Table 1, there are significant differences between the ADHD and non-ADHD groups across key covariates, such as median age (45.6 years vs. 56.9 years), obesity rate (17.3% vs. 6.4%), and the prevalence of various psychiatric comorbidities. The manuscript only provides descriptive statistics and lacks a systematic assessment of the comparability between the groups.

Response: We agree with the reviewer that it would be informative to add a systematic assessment of the comparability between groups on key covariates. We have therefore now revised Table 1 to include the p-values for group differences regarding these covariates. The p-values were calculated using the Wilcoxon rank-sum test for continuous variables and the chi-square test for categorical variables. Additionally, because the p value is sensitive to sample size, we further calculated standardized mean differences (SMDs) for all baseline covariates to quantify the baseline imbalance between the ADHD and non-ADHD groups.

We have revised the text in the Methods and Results sections accordingly:

Page 8, lines 163-165: *“Covariate balance between the ADHD and non-ADHD groups was assessed using standardized mean differences (SMDs), with p values additionally reported to describe statistical differences between groups using the Wilcoxon rank-sum test and chi-square tests.”*

Page 10, lines 215-217: *“Substantial imbalance in baseline covariates was observed between the ADHD and non-ADHD groups (Table 1), with the exception of sex and CVD; most differences were statistically significant.”*

2. The sample size ratio between the exposed group (ADHD, n = 1,204) and the non-exposed group (n = 79,403) is approximately 1:66. With such significant imbalances and clear baseline differences, relying on traditional multivariate regression for covariate adjustment may be difficult to adequately eliminate residual confounding, especially for cardiometabolic measures closely related to age and obesity rates.

Response: We thank the reviewer for this insightful comment regarding the imbalance in sample size and baseline characteristics between the ADHD and non-ADHD groups and the potential for residual confounding.

In the original analysis, we dealt with these imbalances through traditional adjustment for a broad range of covariates, including sex, calendar year and age at T2D diagnosis, psychiatric disorder history, and somatic comorbidities. In sensitivity analyses, we further improved comparability by excluding individuals with a history of schizophrenia, bipolar disorder, or substance use disorder, and by additionally adjusting for psychotropic medication use. The association between ADHD and BMI was attenuated after these additional adjustments but remained statistically significant, suggesting that the findings were not fully explained by measured comorbidities. In stratified analyses we explored the age effects by stratifying by birth cohort, which showed consistent results in the younger cohort where ADHD diagnosis is more routinely captured, suggesting that findings are not solely an artefact of comparing younger ADHD-diagnosis individuals with older non-ADHD individuals.

In addition to conventional multivariable regression, we have now conducted a sensitivity analysis using inverse probability of treatment weighting (IPTW) based on propensity scores to better balance baseline covariates between groups. Additionally, we provide a separate supplementary table (Table S22) showing the SMDs after IPTW. As shown in Table S22, IPTW reduced standardized mean differences across many covariates, including key cardiometabolic factors such as obesity, indicating improved comparability between groups. Additionally, IPTW-weighted analyses yielded results consistent with our primary findings (Tables S23-S25), confirming the robustness of our estimates. The exception was the reduction in BMI post-T2D diagnosis, which was substantially attenuated and no longer statistically significant.

We have revised the manuscript accordingly to improve transparency and explicitly report the additional IPTW analyses in the Methods and Results sections:

Page 9, lines 200-204: *“To further address potential issues of imbalanced covariates between the ADHD and non-ADHD groups, we conducted a sensitivity analysis using inverse probability of treatment weighting (IPTW) based on propensity scores. The propensity scores were estimated using logistic regression including demographic characteristics and relevant psychiatric and somatic comorbidities used in the fully adjusted model, and stabilized weights were used in the analyses.”*

Page 12, lines 280-284: *“Furthermore, after IPTW, the SMDs were reduced, and many covariates were below the conventional threshold of 0.1, indicating improved covariate balance (Table S22). The IPTW-weighted analyses yielded results consistent with the main results (Table S23-S25), in terms of coefficient estimates and overlapping confidence intervals, supporting the robustness of our findings. An exception was the BMI reduction post-T2D diagnosis which was substantially reduced and not significant (β_{ADHD*t}:-0.05 [95%CI,-0.17,0.07]).”*

Taken together, these approaches help mitigate concerns regarding residual confounding due to the imbalance in group sizes. Nevertheless, as with all observational studies, we acknowledge that residual confounding cannot be entirely ruled out and have this clearly stated as a limitation in the Discussion section:

Page 14, lines 342-346: *"We did observe, however, that the reduction in BMI following T2D diagnosis substantially attenuated in IPTW analyses, suggesting that this association is likely driven by differences in baseline characteristics and comorbidities rather than an independent effect of ADHD. Further research is needed to elucidate the underlying mechanisms and disentangle potential sources of confounding."*

Page 16, lines 379-386: *"Fourth, given the observational nature of this study, residual confounding cannot be excluded. However, our sensitivity analyses using IPTW and stratification by birth cohort and sex, generally suggested robustness in the main results. For the longitudinal analyses the greater BMI reduction following T2D diagnosis among individuals with ADHD persisted after adjustment for obesity and baseline BMI, but was attenuated after IPTW adjustment accounting for age, sex, birth year, somatic and psychiatric comorbidities. These findings should therefore be interpreted with caution, as the observed BMI changes may partly reflect differences in baseline characteristics, metabolic risk profiles, and potential for weight loss between groups."*

3. Table 2 showed that the ADHD group had lower systolic blood pressure and higher eGFR. However, the ADHD group was on average over 11 years younger, and age is a strong determinant of both blood pressure and renal function. Although was age is adjusted in the model, the current analysis may not completely peel off the impact of age differences on these results, and it is recommended to interpret the results more cautiously.

Response: We thank the reviewer for this important observation regarding the potential impact of age differences on clinical parameters between the ADHD and non-ADHD groups.

We would like to clarify that the mean values of clinical parameters presented in Table 2 are purely descriptive and unadjusted. Therefore, we agree that the observed differences, such as lower systolic blood pressure and higher eGFR in the ADHD group, should not be interpreted as evidence of independent associations, as they may largely reflect the younger age distribution in the ADHD group. Indeed, the associations are not significant when statistically compared and adjusting for age and other covariates.

We have adopted a more cautious interpretation of baseline cardiometabolic profiles in the Results section, and clarified that these are descriptive:

Page 10, lines 227-233: *"**Descriptive statistics indicated that** at first recorded T2D diagnosis, the ADHD group exhibited a slightly more detrimental cardiometabolic profile, including higher levels of HbA1c, diastolic blood pressure, BMI, total cholesterol, triglycerides and LDLc, and lower levels of HDLc compared to the non-ADHD group. Compared to the non-ADHD group, a higher proportion of individuals with ADHD were*

smokers (36% vs. 21%) and reported low physical activity levels (55% vs. 48%) at T2D diagnosis (Table 2). Conversely, the ADHD group exhibited lower mean systolic blood pressure and higher eGFR levels, which are indicators of more favourable cardiometabolic profiles (Table 2).”

4. Table 1 shows that the obesity rate at T2D diagnosis was significantly higher in the ADHD group compared to the non-ADHD group (17.3% vs. 6.4%). Individuals with a higher baseline BMI or obesity rate often have greater potential for reduction. Although baseline BMI was adjusted for in a sensitivity analysis (Table S10), such a marked baseline disparity suggests the two groups originated from different metabolic risk structures and clinical management contexts. The authors need to be more cautious when interpreting longitudinal BMI changes.

Response: We thank the reviewer for this important comment regarding the substantial difference in obesity prevalence at baseline between the ADHD and non-ADHD groups. We agree that the higher prevalence of obesity in the ADHD group (17.3% vs. 6.4%) shows that the two groups differ in their underlying metabolic risk profiles and clinical contexts, and that individuals with higher baseline BMI may have greater potential for subsequent reduction.

To address this concern, baseline BMI (and obesity status) was accounted for in our analyses. Specifically, we adjusted for baseline BMI in sensitivity analyses (Table S10), and the results remained consistent with the primary findings. In addition, we have now further applied inverse probability of treatment weighting (IPTW) based on propensity scores to improve the balance of baseline characteristics, including obesity, between groups.

Nevertheless, we acknowledge that residual differences in underlying metabolic risk structures and clinical profiles may not be fully captured, and thus the observed longitudinal changes in BMI should be interpreted with caution. We have revised the Discussion section to reflect a more cautious interpretation of these findings:

Page 16, lines 379-386: *“Fourth, given the observational nature of this study, residual confounding cannot be excluded. However, our sensitivity analyses using IPTW and stratification by birth cohort and sex, generally suggested robustness in the main results.* ***For the longitudinal analyses the greater BMI reduction following T2D diagnosis among individuals with ADHD persisted after adjustment for obesity and baseline BMI, but was attenuated after IPTW adjustment accounting for age, sex, birth year, somatic and psychiatric comorbidities. These findings should therefore be interpreted with caution, as the observed BMI changes may partly reflect differences in baseline characteristics, metabolic risk profiles, and potential for weight loss between groups.”***

5. The study required that all metabolic measurements be completed within one month after T2D diagnosis (lines 113-114). This requirement poses a higher

threshold for individuals with ADHD, who may experience challenges with executive function and organization. This could lead to the selected ADHD cohort being a subgroup with better adherence and potentially less severe conditions, affecting the sample's representativeness and the validity of baseline comparisons. This potential bias has not been discussed in the manuscript.

Response: We thank the reviewer for this thoughtful comment regarding the potential for selection bias introduced by requiring baseline measurements within one month of T2D diagnosis.

First, we would like to clarify that our focus is on measurements at T2D diagnosis (i.e., baseline), thus, restricting baseline measurements to within one month of diagnosis allowed us to maximize the sample size while ensuring that clinical parameters closely reflected the physiological status at the time of diagnosis. Expanding this window might increase measurement heterogeneity and reduce the accuracy of baseline comparisons.

Second, our data do not suggest reduced follow-up or measurement frequency in the ADHD group. As shown in Table S3, the median number of clinical measurements during follow-up was comparable between the ADHD and non-ADHD groups. Furthermore, Figure S3 indicates that individuals with ADHD tended to receive follow-up measurements earlier rather than later. For example, among individuals with at least three BMI measurements, the median time to the third measurement was 1.0 years in the ADHD group and 1.3 years in the non-ADHD group. These findings suggest that individuals with ADHD were less likely to be underrepresented due to reduced healthcare engagement and may, in fact, have received more timely follow-up in our sample.

However, we do not have data on individuals with ADHD who were not measured or on ADHD severity. We acknowledge that some degree of selection bias cannot be entirely excluded. As the reviewer pointed out, it remains possible that individuals with more severe ADHD symptoms are less likely to undergo clinical measurements. Therefore, we have explicitly stated in the Discussion section:

Page 15, lines 368-372: *"First, register data only captures individuals who visited healthcare facilities, potentially overrepresenting those with more severe conditions. While it is also possible that individuals with more severe ADHD symptoms may be less likely to undergo clinical measurements and may have poorer metabolic profiles, the underrepresentation of such individuals might contribute to more conservative estimates in our study."*

Reviewer: 2
Dr. Seigo Okada, Yamaguchi University School of Medicine Graduate School of Medicine

Comments to the Author:

This manuscript examines the association between attention-deficit/hyperactivity disorder (ADHD) and cardiometabolic risk profiles among adults with newly diagnosed type 2 diabetes (T2D) using Swedish nationwide register data. The study benefits from a large sample size, high-quality registry data, and extensive sensitivity analyses. The authors have also appropriately acknowledged several methodological strengths and limitations.

However, despite these merits, I have substantial concerns regarding the interpretation of the findings and the validity of the conclusions. In particular, the main conclusions appear to overstate the clinical and conceptual implications of relatively modest and inconsistent associations. While the authors correctly describe the limitations of their data, these limitations are not adequately reflected in the final interpretation. Overall, I am not convinced that the current results sufficiently support the authors' key claims or justify the proposed clinical implications.

While the dataset is large and well curated, the conceptual advance of the study appears limited. The findings largely confirm previously reported associations between ADHD, obesity, and smoking in the general population and do not substantially extend current knowledge regarding T2D management. Given the absence of strong or consistent effects across most cardiometabolic domains, it is unclear whether this study meaningfully changes existing clinical or scientific understanding.

Response: We thank the reviewer for this thoughtful and constructive assessment of our work. We agree that the interpretation and clinical implications of the findings should be aligned with the magnitude and consistency of the observed associations, and that some statements in the previous version may have overstated the broader implications of the results.

We agree that the majority of the cardiometabolic measures were broadly similar between individuals with and without ADHD. Importantly, however, these largely null findings are themselves informative, as they suggest that co-occurring ADHD in adults with T2D is not associated with pervasive cardiometabolic dysregulation across all examined domains. Rather, the pattern observed in our study was more specific, characterised primarily by younger age at T2D diagnosis, somewhat higher BMI, elevated triglycerides, and higher smoking prevalence among individuals with ADHD. While these associations were statistically significant, we acknowledge that the effect sizes were generally modest and should be interpreted accordingly. We have therefore revised the manuscript to more clearly emphasise that the findings should not be interpreted as evidence of broadly worse cardiometabolic control among adults with ADHD and T2D, and to provide a more cautious and balanced interpretation throughout.

Meanwhile, we believe the study contributes novel information by providing the first nationwide description of cardiometabolic profile and longitudinal analysis of cardiometabolic trajectories among adults with co-occurring ADHD and T2D. Our study aimed to characterise the cardiometabolic risk patterns at T2D diagnosis and

longitudinally thereafter in a large nationwide cohort of adults with co-occurring ADHD and T2D, a population that has been very limitedly studied previously. Although the observed differences were modest, we believe some findings may still be clinically relevant from a preventive and monitoring perspective. In particular, the substantially younger age at T2D diagnosis and higher prevalence of smoking and obesity-related measures among adults with ADHD may indicate an earlier accumulation of metabolic risk factors in this group.

In response to the reviewer's comments, we have revised the manuscript throughout in Abstract, Discussion and Conclusion sections to better reflect the modest magnitude of the associations and the predominance of similar cardiometabolic profiles across group, and clarify the contribution to the field, while avoiding overstatement of the clinical implications:

Page 2, lines 44-50: *"At T2D diagnosis, individuals with ADHD were younger and had statistically significantly higher body mass index (BMI) (β_{ADHD}:1.93 [95%CI:1.54,2.32]), triglycerides (β_{ADHD}:0.31 [95%CI:0.17,0.45]) and smoking prevalence (RR:1.58 [95%CI:1.44,1.74]) compared with those without ADHD.* ***Other cardiometabolic risk factors did not differ significantly.*** *Over the subsequent five years,* ***trajectories in cardiometabolic risk factors were broadly similar****, except for a greater reduction in BMI in individuals with ADHD (β_{ADHD*t}:-0.26 [95%CI:-0.34,-0.17]), independent of baseline BMI and glucose-lowering medications."*

Page 2-3, lines 53-58: *"Among adults with newly diagnosed T2D,* ***those with co-occurring ADHD had broadly similar cardiometabolic profiles to those without ADHD****, but presented at a younger age with modestly higher BMI, triglycerides, and smoking prevalence. Individuals with ADHD also showed a slightly greater reduction in BMI over time following T2D diagnosis.* ***Despite modest overall cardiometabolic differences, incorporating ADHD status into preventive diabetes care may help identify a younger, more vulnerable subgroup who could benefit from targeted risk factor management."***

Page 12-13, lines 292-295: *"Despite modest overall cardiometabolic differences between individuals with and without ADHD, incorporating ADHD status into preventive diabetes care may help clinicians identify a younger, more vulnerable subgroup who could benefit from targeted cardiometabolic risk factor management."*

Page 16, lines 379-386: *"Fourth, given the observational nature of this study, residual confounding cannot be excluded. However, our sensitivity analyses using IPTW and stratification by birth cohort and sex, generally suggested robustness in the main results.* ***For the longitudinal analyses the greater BMI reduction following T2D diagnosis among individuals with ADHD persisted after adjustment for obesity and baseline BMI, but was attenuated after IPTW adjustment accounting for age, sex, birth year, somatic and psychiatric comorbidities. These findings should therefore be interpreted with caution, as the observed BMI changes may partly reflect differences in baseline characteristics, metabolic risk profiles, and potential for weight loss between groups."***

Page 16, lines 397-401: *<u>“While cardiometabolic profiles were overall similar between groups, if replicated, these findings suggest that incorporating ADHD awareness into prediabetes and diabetes care may facilitate earlier clinical attention directed toward weight management, blood lipid control, and smoking cessation. Such an approach would be particularly relevant for identifying a younger and more vulnerable subgroup who may present with an elevated initial cardiometabolic risk profile at T2D diagnosis.”</u>*

Reviewer: 3
Ms. Mitsuko Nakata, Kyoto Prefectural University of Medicine

Comments to the Author:
This is a well-structured, large-scale study that clearly delineates the impact of ADHD on biomarkers and lifestyles at the time of diagnosis as well as various clinical indicators following the initiation of treatment. I have several concerns from a statistical perspective that I would like the authors to address. This review focuses on the statistical methods and analyses described in the manuscript. I have not reviewed the scientific content related to the topic itself, as it falls outside my field of expertise.

【major】
1. Why is the significance level set so low at 0.0001(One-five-hundredth of the conventional significance level)? Please explain.

Response: We thank the reviewer for this important comment. As described in the Methods section (lines 180-181), the stringent significance threshold of 0.0001 was chosen, which accounts for the large number of statistical comparisons performed in the study and reduce the likelihood of false positive findings arising from multiple testing, using the Benjamini-Hochberg false discovery rate (FDR) procedure. Specifically, the main analyses included 72 statistical models assessing associations between ADHD and multiple cardiometabolic risk factors at and following T2D diagnosis. While it may appear counterintuitive that the adaptive Benjamini-Hochberg procedure yielded a lower operational threshold than the rigid Bonferroni method (0.00069), this is mathematically driven by the extreme significance and clustering of our top-ranked models.

The Benjamini-Hochberg procedure is an adaptive approach that dynamically adjusts its stringency based on the empirical distribution of the observed p values. In our specific dataset, the top 8 ranked models exhibited exceptionally robust associations, with their observed p values all below 0.0001. At the 9th ranked model, however, the observed p value sharply escalated to 0.0065, corresponding to an adjusted p value greater than 0.05, immediately violating the sequential FDR critical threshold for that specific rank. Consequently, the Benjamini-Hochberg procedure truncated the significant findings exactly at the 8th position, thereby setting the operational p value threshold for final significance at 0.0001.

To improve clarity, we have revised the Methods section to further explain the rationale for applying the FDR-adjusted significance threshold:

Page 8-9, lines 181-183: *"A false discovery rate adjusted p-value threshold of 0.0001, generated using the Benjamini-Hochberg procedure*[40] ***was used to infer statistical significance to account for multiple testing across the 72 models in the main analyses and reduce likelihood of false positive findings."***

2. Please provide a clearer description of the GEE model specifications in the Statistical Analysis section, including the choice of the correlation structure and the inclusion of the interaction term between ADHD and time. Especially, please specify how time was modeled: was the linear regression fitted against the number of post-diagnosis measurements or the actual number of years elapsed?

Response: Thank you for this insightful question. We have provided a detailed explanation below regarding the model specifications and the treatment of the time variable.

First, we would like to clarify that in our model, time was modelled as a continuous variable representing the actual number of years elapsed since the T2D diagnosis. This allows the model to account for the irregular intervals between clinical visits in the real-world registry data. This approach estimates the annual rate of change in clinical parameters, providing a more clinically meaningful interpretation of trajectories for clinical parameters over the disease course.

Second, the interaction term is defined between ADHD and time (years) since T2D diagnosis, which was included to test the hypothesis that the longitudinal trajectory of the clinical parameters differs between patients with and without ADHD. The interaction term specifically estimates the difference in the slopes of the clinical parameters over time between the ADHD and non-ADHD groups. A significant interaction indicates that the rate of change in the clinical parameters over the years is modified by the presence of ADHD.

Overall, the specified regression equation (Model 1) was:

$$E(Y_{ij}) = \beta_0 + \beta_1 \cdot ADHD_i + \beta_2 \cdot Time_{ij} + \beta_3 \cdot (ADHD_i \times Time_{ij}) + \beta_4 \cdot Age_i + \beta_5 \cdot Sex_i + \beta_6 \cdot Year_i$$

where *i* indexes individuals and *j* indexes repeated measurements within individual *i*. $E(Y_{ij})$ represents the expected level of clinical parameters. β_3 represents the additional change in clinical parameter over time (years) since T2D diagnosis associated with ADHD, corresponding to the interaction term.

Finally, in our GEE model, we employed an exchangeable correlation structure to account for within-individual correlations across repeated measurements following T2D diagnosis. This structure assumes that the correlation between any two observations from the same individual is constant, regardless of the time interval between them. We selected this correlation structure because the study aimed to estimate population-averaged longitudinal changes, and repeated measurements were obtained from

routine clinical practice with varying follow-up intervals and numbers of observations per individual, making a more restrictive time-dependent correlation structure less appropriate.

We have clarified the above-mentioned points in the Method section:

Page 8, lines 170-175: *"An interaction term between ADHD and* ***time (years) since T2D diagnosis*** *(β_{ADHD*t}) was included to estimate differences in annual rates of change over follow-up between groups.* ***Time was modelled as a continuous variable to accommodate irregular measurement intervals. An exchangeable correlation structure accounted for within-individual correlations across repeated measurements, varying follow-up intervals and numbers of observations per individual, to estimate population-averaged longitudinal changes."***

3. Were non-linear models considered for the relationship between time and BMI changes? Please provide more details in the Statistical Analysis section.

Response: We thank the reviewer for this thoughtful suggestion. In the present study, time since T2D diagnosis was modelled as a continuous linear term in the GEE analyses. We did not additionally model non-linear time effects because the primary objective was to estimate the average population-level longitudinal difference in BMI changes between individuals with and without ADHD over the follow-up period, rather than to characterize detailed individual BMI trajectories.

In addition, the repeated measurements were derived from routine clinical care in register data, resulting in irregular follow-up intervals and varying numbers of observations across individuals. Under these real-world data conditions, more flexible non-linear specifications may be difficult to interpret and potentially sensitive to sparse measurements at later follow-up periods. Therefore, we considered a linear time specification to provide a parsimonious and interpretable summary of the average population-level longitudinal change in BMI.

To improve clarity, we have revised the Statistical Analysis section to explicitly describe the rationale of not using non-linear models in our study:

Page 8, lines 175-176: *"Non-linear time effects were not modelled, as the irregular timing and frequency of routine clinical measurements supported a parsimonious linear specification."*

【minor】

Line 238: Based on Table S5, the beta coefficients for females appear to be smaller than those for males. Could you please verify this?

Response: We thank the reviewer for this careful observation. After re-examining the estimates presented in Table S5, we can verify that indeed the effect size is larger (more negative) in females, however, with the smaller sample size than for males, the confidence intervals (CIs) are wider and the effect is not significant.

Specifically, for longitudinal BMI change, the estimated interaction coefficient was -0.22 (95% CI, -0.31, -0.13) among males and -0.31 (95% CI, -0.47, -0.15) among females. As more negative coefficients indicate greater reductions in BMI (related to ADHD) over time, the point estimate was numerically larger among females rather than males. However, the confidence intervals for males and females overlapped substantially, therefore, these findings should not be interpreted as evidence of a statistically meaningful sex difference in longitudinal BMI trajectories.

To address this issue, we have revised the Results section to provide a more accurate and cautious interpretation of the sex-stratified findings, as we realize that the phrasing in the previous version could be misinterpreted:

Page 11, lines 257-259: *"In the longitudinal analysis, statistically significant reduction in BMI over time was observed in males with ADHD (β_{ADHD*t}: -0.22 [95%CI, -0.31,-0.13];Table S5). Similar BMI longitudinal patterns were observed in females, however these were not statistically significant (β_{ADHD*t}: -0.31 [95%CI, -0.47,-0.15];Table S5)."*

Line 334: Please also discuss the imbalance in birth cohorts observed in Table 1.

Response: We thank the reviewer for this important observation. Individuals with ADHD were indeed disproportionately represented in the younger birth cohort (1960-2002) compared with the older cohort (1930-1959). This pattern reflects both the historical underdiagnoses of ADHD in older generations and the substantial increase in awareness, diagnostic practices, and access to ADHD treatment over recent decades. We have further clarified in the Discussion section in the manuscript:

Page 15-16, lines 375-377: *"Third, register coverage, diagnostic practices and awareness of ADHD have improved over the past decades,[51] reflected in increased ADHD diagnoses in the younger cohort and a subsequent overrepresentation of younger individuals within the ADHD group (Table S26)."*

To address potential confounding by birth cohort, all primary analyses were adjusted for birth year (by adjusting for both age and year at baseline), and we have conducted stratified analyses by birth cohort (Tables S4-S5) to further enhance age homogeneity across the comparison groups. Stratification by birth cohort suggested that the higher BMI at T2D diagnosis and greater subsequent BMI reduction among individuals with ADHD were primarily observed in more recent birth cohorts, in whom ADHD diagnosis is likely more completely ascertained. This pattern indicates that the main findings are unlikely to be explained solely by age imbalance or secular changes in ADHD diagnosis. However, ADHD ascertainment in older birth cohorts is likely incomplete, and the smaller number of older individuals with ADHD limits interpretation of null findings in this subgroup.

We have added the following to the Discussion:

Page 15, lines 351-358: *“Stratification by birth cohort suggested that the higher BMI at T2D diagnosis and greater subsequent BMI reduction among individuals with ADHD were primarily observed in younger birth cohorts, in whom ADHD diagnosis is likely more completely ascertained, as ADHD is underdiagnosed among older generations.[50] This pattern indicates that the main findings are unlikely to be explained solely by age imbalance or secular changes in ADHD diagnosis. However, ADHD ascertainment in older birth cohorts is likely incomplete, and the smaller number of older individuals with ADHD limits interpretation of null findings in this subgroup. Future studies are needed to explore the birth cohort effects with greater statistical power, and without solely relying on clinical diagnoses of ADHD in the elderly.”*

VERSION 2 - REVIEW

**Reviewer**	**3**
**Name**	**Nakata, Mitsuko**
**Affiliation**	**Kyoto Prefectural University of Medicine, Department of Biostatistics**
**Date**	**09-Jun-2026**
**COI**	

Thank you for your clear and detailed response to my questions. Below are my comments on major comments 1. All the other issues raised by the reviewers have been successfully resolved. I would like to thank you again for your thorough and constructive feedback.

Major Comments:

1. Please explicitly state in the manuscript that the False Discovery Rate (FDR) threshold was set at less than 0.05.

VERSION 2 - AUTHOR RESPONSE

Reviewer: 3

Ms. Mitsuko Nakata, Kyoto Prefectural University of Medicine
Comments to the Author:
Thank you for your clear and detailed response to my questions. Below are my comments on major comments 1. All the other issues raised by the reviewers have been successfully resolved. I would like to thank you again for your thorough and constructive feedback.

Major Comments:

2. Please explicitly state in the manuscript that the False Discovery Rate (FDR) threshold was set at less than 0.05.

Response: We thank the reviewer for the encouraging feedback. In response to the final comment, we have stated in the Method section that the False Discovery Rate (FDR) threshold was set at less than 0.05:

Page 8-9, lines 182-184: *“A false discovery rate adjusted p-value threshold of 0.0001, generated using the Benjamini-Hochberg procedure,[40] was used to infer statistical significance to account for multiple testing across the 72 models in the main analyses and reduce likelihood of false positive findings.”*
